# A tool supporting person-centred communication among people with cognitive complaints, care partners, and clinicians: a feasibility study

**DOI:** 10.1016/j.pecinn.2026.100504

**Published:** 2026-09-10

**Authors:** Tanja J. de Rijke, Thomas Engelsma, Heleen M.A. Hendriksen, Dianne Vasseur, Huiberdina L. Koek, Joost W. van den Berg, Niki Schoonenboom, Ellen M.A. Smets, Leonie N.C. Visser

**Affiliations:** a Amsterdam UMC Location University of Amsterdam, Department of Medical Psychology, Meibergdreef 9, Amsterdam, the Netherlands; b Amsterdam Public Health Research Institute, Digital Health/Personalised Medicine/Quality of Care/Aging & Later Life, Amsterdam, the Netherlands; c Alzheimer Center Amsterdam, Neurology, Vrije Universiteit Amsterdam, Amsterdam UMC Location VUmc, Amsterdam, the Netherlands; d Amsterdam Neuroscience, Neurodegeneration, Amsterdam, the Netherlands; e Amsterdam UMC location University of Amsterdam, Department of Medical Informatics, eHealth Living & Learning Lab Amsterdam, Meibergdreef 9, Amsterdam, the Netherlands; f Vilans, the Dutch National Centre of Expertise for Care and Support, Utrecht, the Netherlands; g UMC Utrecht, Department of Geriatric Medicine, Utrecht, the Netherlands; h Amsterdam UMC location University of Amsterdam, Department of Internal Medicine, Section Geriatrics, De Boelelaan 1117, Amsterdam, the Netherlands; i Spaarne Gasthuis, Department of Neurology, Haarlem, the Netherlands; j Karolinska Institutet, Division of Clinical Geriatrics, Center for Alzheimer Research, Department of Neurobiology, Care Sciences and Society, Stockholm, Sweden; k UMC Utrecht, Julius Center, Department of Bioethics & Health Humanities, Utrecht, the Netherlands

**Keywords:** Patient-clinician communication, Memory clinic, Person-centred care, Feasibility, Alzheimer, Dementia, User experience

## Abstract

**Objective:**

The tool Helder in Gesprek (HiG; translation ‘Clear in Conversation’) aims to enhance person-centred communication during consultations by supporting people with cognitive complaints to articulate topics they wish to discuss with their clinician. HiG also facilitates preparation at home through the selection of topic cards. This study investigates the feasibility of implementing HiG in a Dutch outpatient care setting.

**Methods:**

We conducted a feasibility study in memory clinics, geriatrics departments, and case manager settings. We recruited seven clinicians who recruited ten people with cognitive complaints with a scheduled visit and seven care partners. Observations were done during consultations (*n* = 12). Afterwards, all participants completed questionnaires and people with cognitive complaints and clinicians were interviewed. Audio-recordings of consultations and interviews were inductively coded and thematically analysed.

**Results:**

Fourteen out of twenty-one topic cards were discussed in consultations. HiG was generally well received, acceptable, and usable. The tool helped people with cognitive complaints to discuss topics they considered important and to feel seen as a person, and clinicians to know them better. People were likely to recommend HiG to others (7.9 ± 1.6). Some participants voiced implementation barriers, e.g., card sorting complexity and uncertainty about the optimal implementation point in patient journeys.

**Discussion and innovation:**

Many topic cards were deemed relevant and addressed in consultations, highlighting the relevance of person-centred communication. This study showed that the concept of HiG is feasible. Future research should address the effectiveness of the tool and implementation thereof in (outpatient) care practice.

**Innovation:**

The tool provides people with cognitive complaints with power for agenda setting and agency over what they want to share with the clinician.

## Highlights

• The tool helps people with cognitive complaints to introduce relevant topics.
• People with cognitive complaints shared personal experiences, needs, and activities.
• The tool helps people with cognitive complaints to feel seen as a person.
• The tool helps clinicians to get to know people with cognitive complaints better.
• This study underscores the need for person-centred communication and care.

## 1 Introduction

Worldwide, the number of people with dementia is expected to rise from 50 to 152 million by 2050 due to an aging population and the lack of effective preventive and/or widely available therapeutic options [1]. An increasing number of people with cognitive problems, including people with dementia, will therefore need healthcare and support services, provided in memory clinics and other (outpatient) care settings [2]. Consequently, attention must be given to how care is delivered and the quality of care in these settings*.* During care consultations, person-centred communication is key, i.e., ensuring attention to the person as a whole, sharing information and decisions, providing compassionate and empowering care, and being sensitive to needs [3], [4], [5], [6], [7]. A person-centred communication approach has a positive impact in various ways. To begin with, it helps clinicians to obtain a comprehensive picture of the person with cognitive complaints and their relative(s), which facilitates the provision of appropriate care [8]. Moreover, attuning communication to people's information needs is also found to positively affect patient outcomes, such as patient satisfaction and overall wellbeing [9], [10], [11]. In addition, addressing emotional needs specifically has been found to improve information recall and increase satisfaction [12]. At the same time, people with cognitive complaints have expressed a need to feel known and understood by clinicians [13]. Addressing this need can be considered especially relevant for people who experience cognitive decline, because of the challenge cognitive decline poses to a person's sense of self and identity [14]. Taken together, stimulating person-centred communication with people with cognitive complaints, including those with dementia, is important both from an ethical and a practical perspective.

Previous studies showed that person-centred communication is still not common practice, warranting improvement [15], [16], [17], [18]. One of the barriers identified in previous research is that people with cognitive complaints and their accompanying relatives (their ‘care partners’) often do not voice their healthcare needs and preferences [15], [16]. To support people with cognitive complaints and their care partners to better express themselves and facilitate person-centred communication during consultations, we developed a tool called ‘Helder in Gesprek’ (literal translation: ‘Clear in Conversation’; see Box 1) [19]. Through a human-centred design approach including co-research and co-design with people with cognitive complaints, care partners, and clinicians, prototypes were developed in both digital and analogue form. The ‘Helder in Gesprek’ tool aims to encourage people with cognitive complaints who visit a healthcare service such as the memory clinic to identify in advance the most important topics they wish to discuss with their clinician and to articulate these topics during the consultation. By stimulating person-centred communication, the tool would help the clinician to gain a more comprehensive understanding of this unique person, their needs, and their life. Ultimately, ‘Helder in Gesprek’ aims to contribute to high-quality, person-centred care.

**Fig. 1:**
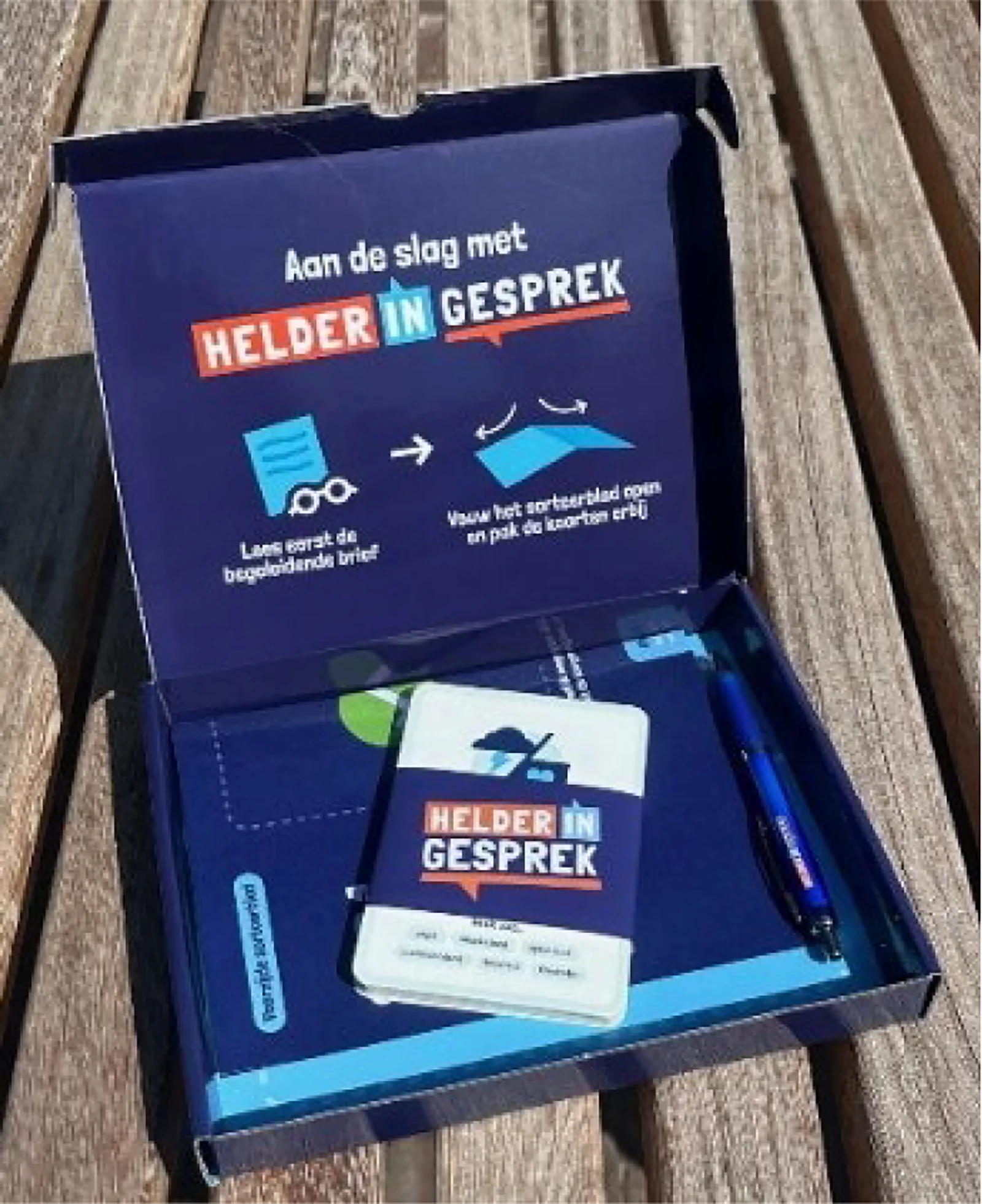

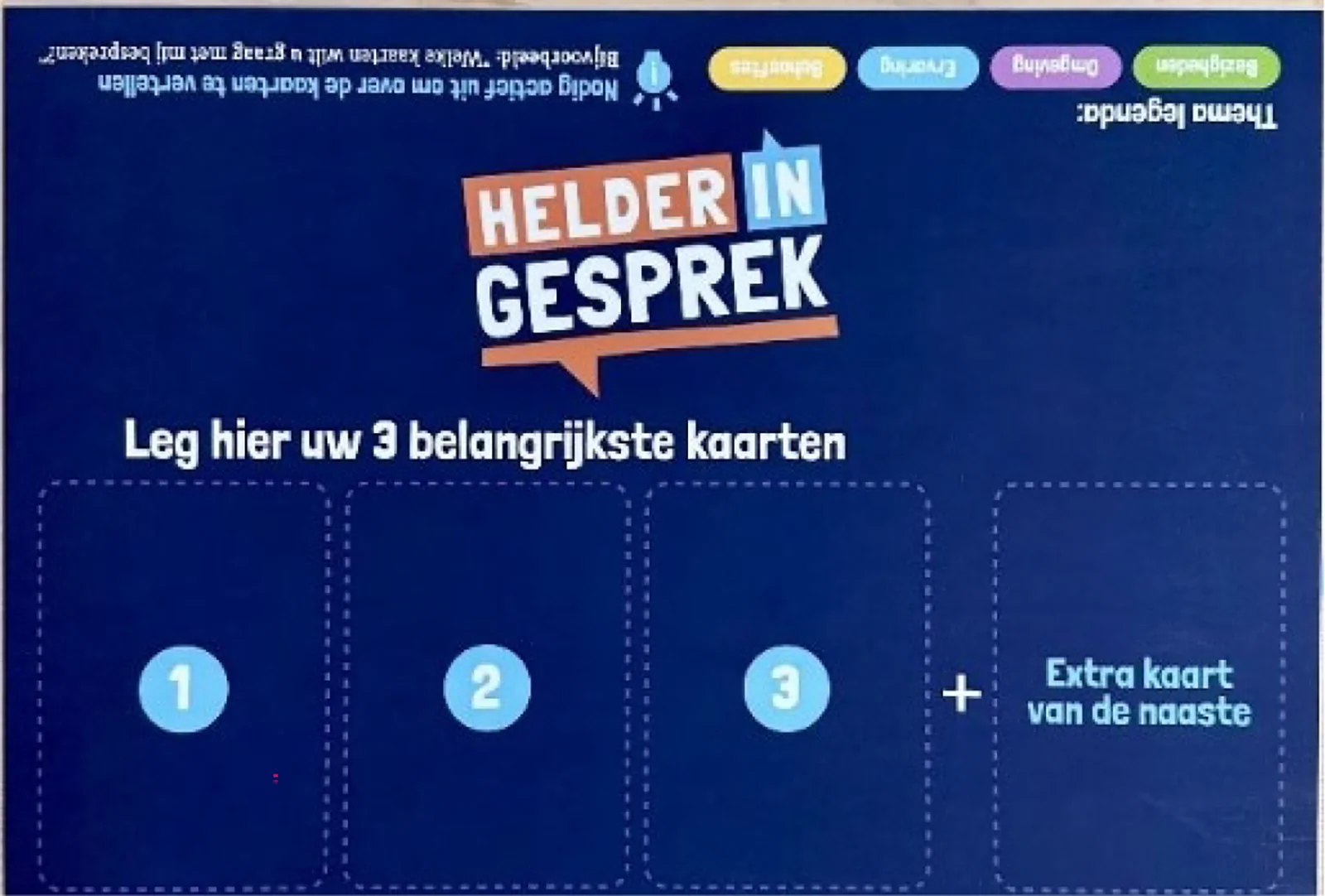

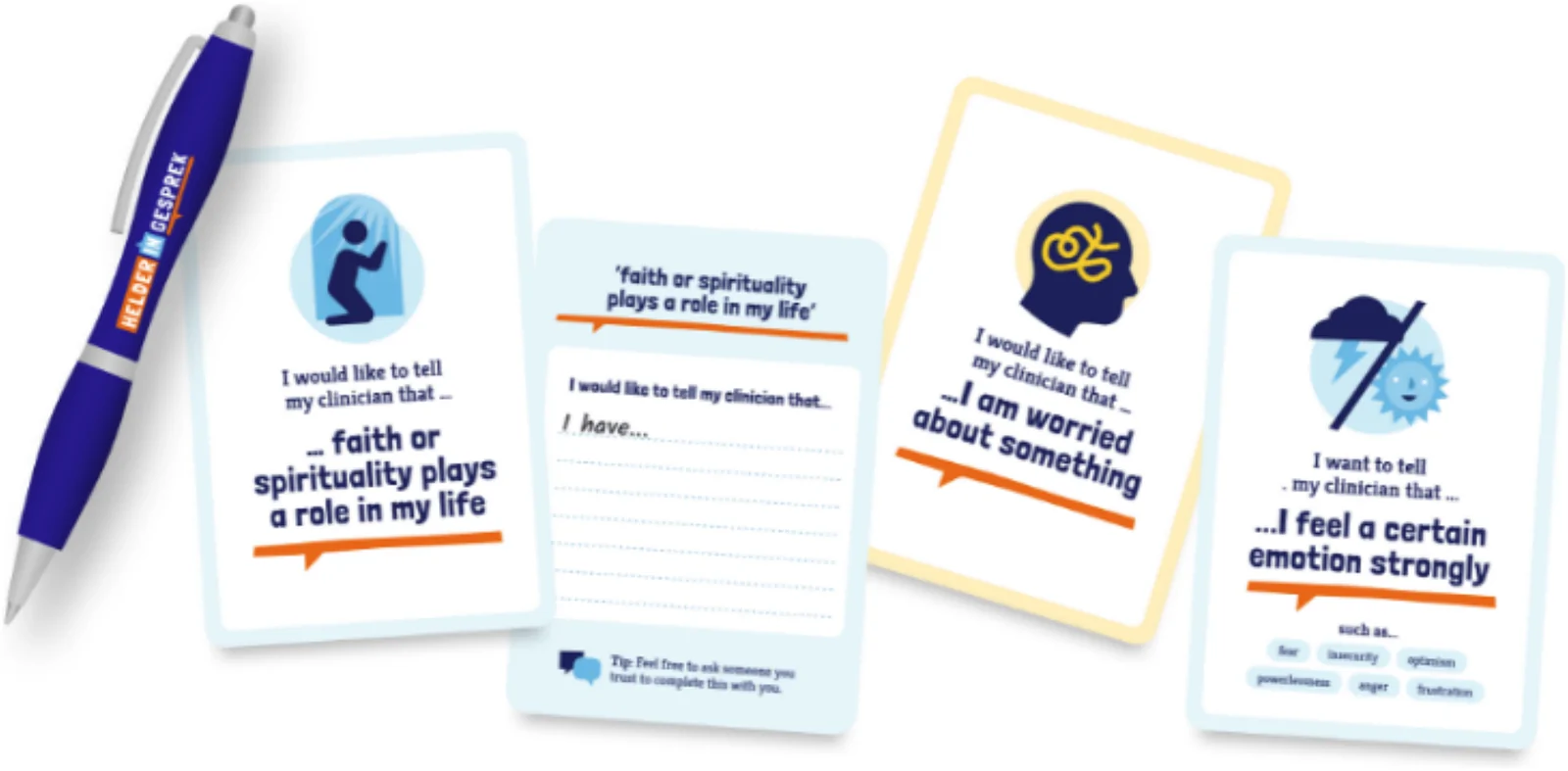
a The ‘Helder in Gesprek’ box that people receive at home. b Placemat for in the consultation room. c Front side of three cards and one reverse side of a card containing space for elaboration.

Box 1 **The ‘Helder in Gesprek’ tool**

‘Helder in Gesprek’ (©2025-ABOARDxAmsterdam UMC) is comprised of a box with cards, a pen, a sorting sheet, and an accompanying letter with brief explanation (see Fig. 1a). The form of a box with cards is directly informed by previous co-research and co-design processes, as described in De Rijke et al. [19]. The box fits in standard sized Dutch mailboxes. The set of cards contains statements all starting with ‘*I would like to tell the clinician that….’*, divided over the themes Activities (e.g. *‘I would like to tell the clinician that I stay at home more than I used to.’*), social environment (e.g. ‘*I would like to tell the clinician that I experience a lot of support from the people around me’*), Experiences (e.g. ‘*I would like to tell the clinician that I feel a certain emotion strongly’),* and Needs (e.g. *‘I would like to tell my clinician that I am worried about something’*) (see Fig. 1b and 1c). All reverse sides of the cards contained space for written elaboration. For the full list of 21 topic cards see appendix 1. The box also contains a blank card to allow people to write down another topic that they would like to discuss. Additionally, another blank card is available for care partners specifically to write down and voice topics they would like to discuss. The sorting sheet asks people to sort the cards into two piles: topics that they would like to discuss with their clinician, and topics that they would not necessarily like to discuss with their clinician. Next, people with cognitive complaints are asked to select a maximum of three cards that they would prefer to address during the upcoming consultation. They are asked to bring these three cards to the consultation.

Evaluations of initial prototypes regarding the usability and user experience (UX) of the ‘Helder in Gesprek’ tool among people with cognitive complaints, care partners, and clinicians identified areas for improvement regarding navigation, system feedback, clarity of content, and cognitive load [19], [20]. These findings informed the re-design of the analogue prototype. We concluded that an analogue tool is more usable for and preferred by people with cognitive complaints and thus more inclusive in comparison to the fully digital prototype, especially among people with dementia, and therefore decided to prioritise the re-design of the analogue prototype. The aim of the current study is to investigate the feasibility of adopting the analogue ‘Helder in Gesprek’ tool in a Dutch real-world outpatient setting among people with cognitive complaints, care partners, and clinicians. Additionally, we aimed to explore whether the tool supports person-centred communication and makes people with cognitive complaints feel more seen, heard, and understood.

## 2 Methods

### 2.1 Study design and study setting

We conducted a cross-sectional, mixed-methods feasibility study. Feasibility research identifies aspects of research methods or procedures that may need adjustment and offers insight on how these changes can be implemented by addressing acceptability, demand, implementation, practicality, adaptation, integration, expansion, and limited efficacy testing [21]. The study was conducted in various outpatient memory clinics and geriatrics departments in the Netherlands, in both academic and non-academic hospitals, and dementia case manager settings. Memory clinics are multidisciplinary clinics that aim to offer early and timely care and support for people with cognitive complaints [22]. Syndrome diagnoses or diagnostic labels determined at the memory clinic comprise dementia, mild cognitive impairment (MCI), which objectively shows in cognitive testing without losing independency in daily living, and subjective cognitive decline (SCD), which does not objectively show in cognitive testing [23]. Geriatrics departments are dedicated to addressing the distinct healthcare needs of older adults, with a particular emphasis on the management of complex, multimorbid conditions, and aims to enhance quality of life, support functional independence, and promote healthy aging through holistic care, typically delivered by a multidisciplinary team [24], [25], [26]. In this study, memory clinics were either part of a neurology department, hereafter referred to as memory clinic, or a geriatric department, hereafter referred to as geriatric department. A dementia case manager is a healthcare professional, usually a specialized nurse, who guides and supports people with dementia and their care partners at home after the diagnosis. Case managers may assist with the organization of care and support, provide information, and act as a point of contact for people with dementia and their care partners. In the Netherlands, dementia case managers usually do not work in a memory clinic, but rather for care and welfare organisations or specialized regional dementia networks. Data collection ran from July 2025 until November 2025, as part of the ABOARD project (www.aboard-project.nl). The acronym ABOARD stands for A *Personalized Medicine Approach for Alzheimer's Disease*, aiming to ensure a future of personalized, patient-orchestrated diagnosis, prediction, and prevention of Alzheimer's disease (AD) [27]. The Medical Ethics Committee of the Amsterdam UMC, location AMC, approved the study (2025.0513). All participants provided informed consent prior to collecting any data. The study complies with ethical standards of the Helsinki Declaration [28].

### 2.2 Study participants and recruitment

#### 2.2.1 Clinicians

The objective was to recruit ten clinicians, who would then each recruit approximately five people with cognitive complaints scheduled for a consultation and, if present and willing to participate, their accompanying care partners. Clinicians needed to work in an outpatient care setting for people with cognitive complaints, for a dementia-related diagnostic work up and/or provision of care/support, including neurologists, geriatricians, (specialized) nurses, neuropsychologists, and case managers. We aimed for variation between clinicians in terms of type of hospital and profession. Clinicians were recruited using convenience sampling via our extensive network, a LinkedIn post, and newsletters (e.g. Dutch Memory Clinic Network). Interested clinicians were contacted per e-mail, followed up by a short information session (one-on-one or with a few clinicians at once) during which information was provided about ‘Helder in Gesprek’ and the feasibility study. Participating clinicians provided informed consent and received further instructions from the researcher (TR; MSc; PhD student; female; trained in qualitative research) during a one-hour face-to-face meeting.

#### 2.2.2 People with cognitive complaints and care partners

Inclusion criteria for people with cognitive complaints and care partners comprised having a scheduled consultation with a participating clinician in the upcoming four weeks because of cognitive complaints, being able to formulate opinions independently, speaking Dutch, and being older than 18 years. Clinicians determined whether people with cognitive complaints were eligible to participate and asked for permission for the researcher to contact them. When deemed eligible and after approval, the researcher (TR) phoned interested people with cognitive complaints to explain the study in more detail and to answer any questions prior to participants providing written or digital informed consent.

People with cognitive complaints, care partners, and clinicians all had a separate option in the informed consent to approve or decline the audio recording of their consultation(s). Once the informed consent was signed, ‘Helder in Gesprek’ was sent to people's home address by post, prior to their consultation (see Box 1. and Fig. 1 for an explanation of ‘Helder in Gesprek’).

### 2.3 Assessments

#### 2.3.1 Implementation strategies

Prior to their first consultation with a participating person with cognitive complaints and/or care partner, participating clinicians received a short, animated video via e-mail containing a brief explanation of ‘Helder in Gesprek’ and instructions regarding their role, i.e., ‘invite the person with cognitive complaints to put the cards on the table and to elaborate on (one of) the cards’. If feasible, a short, animated video was also shown on screens in the waiting room, with the main aim of reminding people with cognitive complaints of ‘Helder in Gesprek’ and empowering them to use the cards and discuss what they considered important. When demonstration on screen was impossible, the video was sent to people with cognitive complaints and care partners via e-mail or shown on a mobile phone in the consultation room by TR (depending on the preference of the participant). If possible, a poster about ‘Helder in Gesprek’ was displayed in the waiting room and/or the consultation room, with the main aim of reminding of ‘Helder in Gesprek’.

In the consultation room, a placemat (or: ‘table mat’) similar to the sorting sheet in the ‘Helder in Gesprek’ box was placed on the desk (see Fig. 1b) with the aim to provide a visual reminder to people with cognitive complaints and care partners to literally put their cards on the table and for clinicians to actively invite them to express what is important to them. The placemat also includes instructions to invite people with cognitive complaints to place their cards and to elaborate on them, and provides clinicians with an example question.

#### 2.3.2 Consultation observations

During the consultation, a researcher (TR) was present to make structured observations and to start and stop the audio-recording, using an encrypted Philips Voice Recorder app (v3.7.0) [29]. For the observations, we had a predefined observation scheme (see appendix 2) to ensure consistency in data collection [30]. Observations comprised which cards were addressed, when they were addressed during the consultation (time stamp), by whom the conversation about the cards was initiated, and the amount of time that was spend on discussing the cards (in relation to the length of the entire consultation).

#### 2.3.3 People with cognitive complaints and care partner evaluation

Directly after their consultation, people with cognitive complaints completed a short supported questionnaire with closed questions on demographics (see appendix 3). Supported meant that their care partner or the researcher (depending on people's preference) read the questions in the questionnaire aloud and could assist in noting down the person's answers. Next, after brief rapport building, the researcher (TR) conducted a short semi-structured interview using open-ended questions asking people with cognitive complaints about their opinions on ‘Helder in Gesprek’ and their evaluation of the consultation (max. 20 min; see appendix 4 for interview guide). This interview was also audio recorded. The care partner was allowed to be present for support, if preferred. Participating care partners reported their opinions regarding ‘Helder in Gesprek’ and evaluation of the consultation, using a questionnaire instead of a semi-structured interview (see appendix 5). All questionnaires and the semi-structured interviews were preferably completed immediately after the consultation. However, if preferred, the questionnaires could be completed at home (on paper or digitally) and people with cognitive complaints could be interviewed by telephone (resulting in approximately half of our sample that preferred to complete the questionnaire and/or conduct the interview by phone at home).

#### 2.3.4 Clinician evaluation

Immediately after each study consultation, clinicians completed one multiple choice and one open-ended question, to capture their experience: 1) their initial impression of whether ‘Helder in Gesprek’ had helped them to get to know this specific person, and 2) what had been helpful, or not, in the use of ‘Helder in Gesprek’ with this specific person (max. 2 min; see appendix 6). After clinicians completed data collection, a closing interview was held (with TR) to reflect on their overall experience with ‘Helder in Gesprek’ and on the feasibility of implementation of this tool in their clinical practice (max. 30 min; see appendix 7).

### 2.4 Data analysis

Descriptive statistics were derived using IBM SPSS version 28 [31]. In case of missing data, we did not exclude any other data collected or input data, yet we do provide insight on potentially missing data points when reporting results by providing the N. Audio-recordings from both the observations and the interviews were transcribed clean verbatim and analysed using MAXQDA [32]. For the qualitative analysis of the observations and interviews, we used a similar approach, first identifying relevant elements by selecting coding units from the interview transcripts and written responses. Coding units were defined as the smallest text segments that carried independent meaning [33]. In this study, a coding unit was any part of a sentence that described: a topic related to ‘Helder in Gesprek’ or a topic that seems to be discussed due to the use of ‘Helder in Gesprek’. After this, we carried out inductive coding to group the elements into more specific categories, applying in vivo coding, axial coding, and subcoding [34]. All consults and interviews were coded independently by TR and a random sample was checked by TE, who then compared their results and resolved differences through discussion until agreement was reached. The finalized coding trees are presented in appendix 8. Finally, we applied thematic analysis to the codes and subcodes in order to identify recurring patterns of meaning (themes), which were discussed with all co-authors for confirmation and refinement purposes. Prior to manuscript writing, TR translated quotes, themes, and categories flowing from the data-analysis from Dutch into English, which was checked by the study team (TE,ES, LV). For reporting, we used the Consolidated Criteria for Reporting Qualitative Research (COREQ; see appendix 9) [35], [36].

## 3 Results

### 3.1 Study sample and flow

Initially, thirteen clinicians expressed interest to participate in the study after receiving the information, of which eleven signed informed consent, and seven were able to successfully recruit one or more people with cognitive complaints and/or care partners for this study during the study data collection period. Characteristics of these seven clinicians are presented in Table 1. Clinicians were mainly female (6/7), 44.6 years old (SD = 6.9), (a type of) nurse (5/7), and had on average 7.8 years work experience (SD = 7.8) in a memory clinic or outpatient care setting (see Table 1).

**Table 1:** Study sample characteristics of clinicians.

	Clinicians (*n* = 7)
Gender (%)	Female: 6/7 (86%) Male: 1/7 (14%)
Age (mean ± SD; in years)	44.6 ± 6.9 (min = 34; max = 52)
What is your profession? (%)*	• Nurse: 5/7 (71%) • Neurologist: 1/7 (14%) • Case manager: 1/7 (14%)
How many years of work experience do you have in the outpatient clinic setting? (mean ± SD)	7.8 ± 7.3 (mi*n* = 1; max = 17)
How many new patients do you see on average per week? (mean ± SD)	6.3 ± 4.3 (min = 1; max = 12)
How many consultations do you have on average with a patient? (mean ± SD)	3.6 ± 3.8 (min = 1; max = 10)
How long does a consultation with a patient last on average in minutes? (mean ± SD)	50 ± 22.9 (min = 20; max = 90)
How long does a (diagnostic) patient journey on average last in weeks? (mean ± SD)**	3.3 ± 1.9 (min = 2; max = 6)

Notes. *comprising of n = 1 nurse, n = 1 specialized nurse, and n = 3 nurse consultants. **n = 3 missings due to the fact that this question is not relevant to the clinical setting of that particular clinician.

After an initial explanation of the study by the clinician, sixteen people with cognitive complaints and eleven care partners were approached by TR. Initially, fifteen people with cognitive complaints and eleven care partners provided verbal informed consent for the study and received the tool at home. Eventually, six people with cognitive complaints and four care partners did not provide written informed consent for study participation after all or declined participation. Reasons for not wanting to participate were mainly that participating in research was considered too stressful or overwhelming (mentioned by *n* = 4 people with cognitive complaints; *n* = 3 care partners). Other reasons comprised: not wanting the consultation to be observed or audio-recorded (*n* = 1 person with cognitive complaints), or considering the topics addressed by ‘Helder in Gesprek’ not relevant and fear for being asked too personal questions (*n* = 1 person with cognitive complaints).

In total, we observed twelve consultations. These comprised ten consultations with ten participating people with cognitive complaints, with five accompanying and participating care partners, and two consultations with care partners only. Participating people with cognitive complaints were mostly female (6/10), 69.6 years old (SD = 8.4), born in the Netherlands (9/10) and had a diverse background when it comes to educational attainment and syndrome diagnosis/diagnostic label (see Table 2). Care partners were mainly partners of the person with cognitive complaints (5/7; see Table 2).

**Table 2:** Study sample characteristics of people with cognitive complaints and care partners.

	People with cognitive complaints (*n* = 10)	Care partners (*n* = 7)
What is you relation to your care partner? (%)	n/a	• Partner: 5/7 (71%) • Family member: 1/7 (14%) • Friend: 1/7 (14%)
Gender (%)	Female: 6/10 (60%) Male: 4/10 (40%)	Female: 5/7 (71%) Male: 2/7 (29%)
Age (mean ± SD; in years)	69.6 ± 8.4 (min = 55; max = 82)	64.6 ± 15.7 (min = 37; max = 84)
Diagnosis / diagnostic label (%)	• Dementia: 5/10 (50%) • MCI: 4/10 (40%) • SCD: 1/10 (10%)	n/a
Educational attainment (%)*	Low: 3/10 (30%) Medium: 3/10 (30%) High: 4/10 (40%)	Low: 3/7 (43%) Medium: 3/7 (43%) High: 1/7 (14%)
Country of Birth (%)	Netherlands: 9/10 (90%) Surinam: 1/10 (10%)	Netherlands: 7/7 (100%)
Language spoken at home (%)	Dutch: 10/10 (100%)	Dutch: 7/7 (100%)

Notes. *Measured using the Dutch CBS division by Pleijers and De Vries [37]. 1-item scale from 1 (low educational attainment) to 10 (high educational attainment) based on the Dutch educational system. Classification was done as follows: low (none or elementary school, primary or preparatory vocational education (vso, vmbo-b, vmbo-k, vmbo-g, vmbo-t, mavo, mulo, lts, leao, lhno of meao), lower grades of secondary school of HAVO or VWO, or assistant training (MBO-1)), medium (higher grades of secondary school of HAVO, VWO, HBS, or MMS, basic vocational training (MBO-2), vocational training (MBO-3), and middle management and specialist education (MBO-4)), and high (higher vocational education (HBO, HEAO, or HTS) and university).

### 3.2 Consultation characteristics

Of the twelve consultations, two were exclusively with a care partner (and the clinician). One consultation took place in a case manager setting, eight in a memory clinic, and three in a specialized geriatric setting. Consultations were mainly follow-up consultations (7/12). In most of the consultations (10/12), discussing ‘Helder in Gesprek’ topic cards did not result in a delay judged based on the scheduled standard duration. The cards were usually discussed in the middle of the consultation (9/12; i.e. not immediately at the start or at the end of the consultation) (see Table 3).

**Table 3:** Consultation characteristics (*n* = 12)*.

Type of consultation (%)	• First consultation: 4/12 (33.3%) • Result consultation: 1/12 (8.3%) • Follow-up consultation: 7/12 (58.3%)
HiG discussed**	• Yes: 12/12 (100%)
HiG discussed correctly?***	• Yes: 12/12 (100%)
Initiator of the discussion of HiG?	• Person with cognitive complaints: 1/12 (8%) • Care partner: 1/12 (8%) • Clinician: 10/12 (83%)
Moment when HiG is discussed ****	• Immediately at the start of the consultation (initiating phase): 3/12 (25%) • In other parts of the consultation (gathering information, summary, explanation, planning phase): 9/12 (75%)
Reverse side of the HiG cards is filled in	• Yes: 4/12 (33%) • No: 8/12 (67%)
Duration of HiG-related communication	11.78 ± 6.59 min (min = 3; max = 28)
Duration of consultation	35.67 ± 14.86 min (min = 20; max = 60)
Mean percentage of consult time e that related to HiG	35% ± 16% (min = 13.7; max = 58,3)

Notes. *Consultations with people with cognitive complaints (n = 10), some of which were accompanied by care partners (n = 5), or consultations with only care partners (n = 2). **HiG=’Helder in Gesprek’. ***Used correctly is defined as using the HiG cards to explore what is important for the person with cognitive complaints or care partner to share with the clinician and that these topics are (briefly) addressed in the consultation.****Phases in the consultation are based on the adaptations made by Manalastas et al. [38], resulting in a final set of consultation phases: initiating, gathering information, summary, explanation, planning, and closing [38].

In the next sections, we first focus on the observations during consultations, chosen topic cards, and subsequently on the experiences and evaluation of people with cognitive complaints, care partners, and clinicians.

### 3.3 Observed use of ‘Helder in Gesprek’ in consultations

Clinicians usually initiated the discussion of ‘Helder in Gesprek’ (10/12), not immediately at the start, yet somewhat in the beginning of the consultation. Across all twelve consultations, thirty-five instances were observed in which a person with cognitive complaints, care partner, and/or clinician engaged in a conversation directly related to the ‘Helder in Gesprek’ topic cards. Of these thirty-five instances, eleven were initiated by people with cognitive complaints and/or their care partners, typically as a natural continuation of the discussion when transitioning from one card to another. Two participants had selected cards at home but had forgotten to bring them; in these cases, clinicians provided the ‘Helder in Gesprek’ box during the consultation, allowing participants, either alone or with their care partner, to pick the desired cards on the spot. In one consultation, a person with cognitive complaints indicated an inability to elaborate on the selected cards during the session, prompting the care partner to use the cards to communicate discussion points on behalf of their loved one, based on their prior preparation and selection of topic cards at home. Two others with cognitive complaints explicitly mentioned that they skimmed through the cards at home, but did not select nor bring any cards to the consultation because they considered the cards not relevant to them or their situation. During these consultations, clinicians laid out all ‘Helder in Gesprek’ cards on the table and asked these people if they wanted to discuss one of the cards. This did not spark a discussion, resulting in clinicians picking and initiating a discussion on a few cards.

In a few consultations, explicit comments about the value of ‘Helder in Gesprek’ were made:

*‘Then this* [points to HiG cards] *is really great. Because otherwise I would have never brought it up.’* (consult P_3, person with cognitive complaints).

Other explicit comments regarding the usability of ‘Helder in Gesprek’ included that using these topic cards was perceived as easier than writing down discussion topics themselves, that the topics on the provided cards were considered appropriate and relevant, that selecting cards at home was challenging for some participants due to the large number of cards that they deemed relevant, and that it was sometimes difficult to formulate specific questions or topics on the blank care partner card:

*‘Well, I didn't have that much, I found it difficult to write down what you think is important to say, because, well, I have 100,000 examples’* (consult P_3, care partner).

One person with cognitive complaints explicitly mentioned that he would have wanted to receive ‘Helder in Gesprek’ sooner in the care trajectory, namely prior to the first consultation in the memory clinic.

*‘But if I had seen that whole row of cards… then I think, well, last time was actually the first consultation, I would have liked to receive those cards earlier to, let's say, involve you in that.’* (consult P_4, person with cognitive complaints).

### 3.4 Chosen topic cards

In total, fourteen out of twenty-one available ‘Helder in Gesprek’ cards were chosen by one or more people with cognitive complaints or care partners to discuss with their clinician (see Table 4).

**Table 4:** Helder in Gesprek (HiG) cards picked to discuss with the clinician during the consultation (number of people; %).

**Activities**	I would like to tell my clinician that (volunteer) work is important to me. (4/12; 33%)
	I would like to tell my clinician that I am no longer employed. (3/12; 25%)
	I would like to tell my clinician that I enjoy exercise. (3/12; 25%)
	I would like to tell my clinician that I stay at home more than I used to. (2/12; 17%)
	I would like to tell my clinician that I enjoy meeting up with friends and/or family. (2/12; 17%)
	I would like to tell my clinician that faith or spirituality plays a role in my life. (0/12; 0%)
**Needs**	I would like to tell my clinician that I would like to prepare for the future. (4/12; 33%)
	I would like to tell my clinician that I am concerned about something. (3/12; 25%)
	I would like to tell my clinician that I need advice or more information. (3/12; 25%)
	I would like to tell my clinician that a lot is happening in my life. (2/12; 17%)
**Experiences**	I would like to tell my clinician that I feel a certain emotion strongly. (3/12; 25%)
	I would like to tell my clinician that I have experienced something traumatic in my life. (3/12; 25%)
	I would like to tell my clinician that I sometimes find it difficult to ask for help. (2/12; 17%)
	I would like to tell my clinician that I feel good about myself. (2/12; 17%)
	I would like to tell my clinician that I have my own idea about what is wrong with me. (0/12; 0%)
	I would like to tell my clinician that I am reluctant to attend this appointment. (0/12; 0%)
**Social environment**	I would like to tell my clinician that I feel a lot of support from the people around me. (0/12; 0%)
	I would like to tell my clinician that people treat me differently than they used to. (0/12; 0%)
	I would like to tell my clinician that I live with other people. (0/12; 0%)
	I would like to tell my clinician that I am often on my own. (0/12; 0%)
**Other**	There is another topic I would like to discuss, namely … (1/12; 8%)*

Notes. Topics are ranked from most chosen to least chosen and the colour indicates the overarching theme. *Topic comprised someone's family history of dementia.

In line with the chosen topic cards described above, discussions in the observed consultations focussed on the future (e.g. worries about the future), work (e.g. what people miss about their job), daily activities (e.g. reading, physical activity, and driving), relationships with other clinicians (e.g. general practitioner or case manager), social environment (e.g. family or friends with a neurological condition or perceived social support from friends and family), coping (e.g. hiding cognitive complaints, last will preparations as this provides peace), symptoms and other health issues (e.g. being able to handle fewer stimuli or memory complaints), and emotion-related topics (e.g. frustration, worries, grief, or gratitude) (for final coding tree, see appendix 7). Care partners often seemed to write symptom-related topics on their card. The quotes below illustrate some of the ‘Helder in Gesprek’-related communication observed:

*‘Clinician [points to HiG card]: What is the emotion that you feel?*

*Care partner: Eh yes, that is eh frustration or that you cannot do anything about it.’* (Consult N_1, care partner).

‘*Well, I enjoy meeting up with friends and/or family. I really enjoy that. Honestly. Yes, together with the children and my daughters-in-law and grandchildren. Yes, they can make me so happy. All together or separately. Yes, that's really…’* (consult P_3, person with cognitive complaints).

*‘Yes, and sometimes when I look at him, I get emotional… Because, well, I just miss the man who used to be there.’* (Consult N_3, care partner).

*‘I exercise a lot and I'm always busy in the garden, and I'll just keep on doing that. It's time to prune again, so I'll be busy with that too.’* (Consult P_15, person with cognitive complaints).

*‘I wonder how long I can continue to live at home? But that is impossible to say.’* (consult P_13, person with cognitive complaints).

Communication related to ‘Helder in Gesprek’ topics frequently led to discussions concerning specific follow-up actions (observed 10/35 instances in which a topic was raised through a ‘Helder in Gesprek’ card across all consultations), information provision (12/35 instances), and/or acknowledgement of, or reassurance in response to, what was said by the person with cognitive complaints (20/35 instances).

### 3.5 Evaluation of ‘Helder in Gesprek’

#### 3.5.1 People with cognitive complaints

**Fig. 2:**
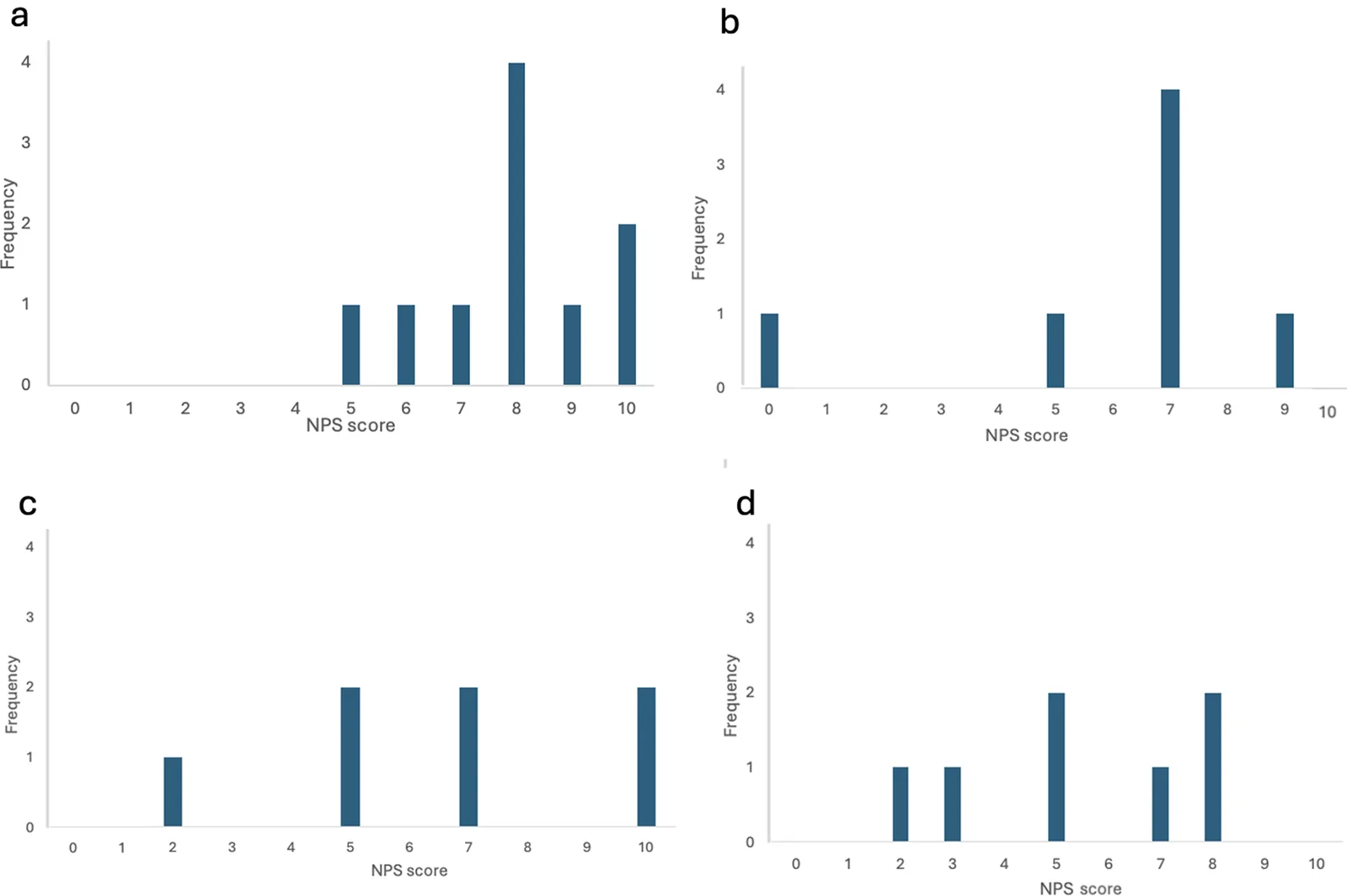
a Likelihood of people with cognitive complaints recommending ‘Helder in Gesprek’ to other people with cognitive complaints. b Likelihood of care partners recommending ‘Helder in Gesprek’ to other care partners. c. Likelihood of clinicians recommending ‘Helder in Gesprek’ to colleagues. d. Likelihood of clinicians recommending ‘Helder in Gesprek’ to people with cognitive complaints. Notes. NPS = Net Promoter Score; 0 = not at all likely and 10 = very likely.

For detailed questionnaire results see Table 5. People with cognitive complaints reported to have used ‘Helder in Gesprek’ at least once at home (10/10), most often together with their partner (6/10) for approximately 16–30 min (6/10). The tool was generally considered easy to use (a little: 2/10; a lot: 4/10; very much: 3/10). In answer to the close-ended questions in the evaluation, most people with cognitive complaints indicated that ‘Helder in Gesprek’ had helped them to discuss topics during the consultation (a little: 5/10; a lot: 3/10). Also most of them reported that their clinician had got to know them as a person (a little: 4/10; a lot: 2/10; very much: 3/10). People were likely to recommend ‘Helder in Gesprek’ to others with cognitive complaints (7.9 ± 1.6; min = 5; max = 10) (see Fig. 2) and a majority planned to use ‘Helder in Gesprek’ during future consultations (somewhat likely: 3/10; very likely: 3/10).

**Table 5:** Evaluation of ‘Helder in Gesprek’ by people with cognitive complaints and care partners.

	People with cognitive complaints (*n* = 10)	Care partners (*n* = 7)
Did you use HiG at home prior to the consultation? (%)	• Yes: 10/10 (100%)* • No: 0/0 (0%) • I do not remember: 0/0 (0%)	• Yes: 5/7 (71%) • No: 1/7 (14%) • I do not remember: 1/7 (14%)
With whom did you use HiG at home? (%)	• Alone: 2/10 (20%) • With my partner: 6/10 (60%) • With a family member: 1/10 (10%) • With my child(ren): 1/10 (10%) • With a friend: 0/10 (0%) • With an acquaintance: 0/10 (0%) • With someone else: 0/10 (0%) • I do not remember: 0/10 (0%)	• With my care partner: 5/7 (71%) • I used HiG on my own: 0/0 (0%) • I used HiG with my care partner and on my own: 0/0 (0%) • I did not use HiG: 1/7 (14%) • Other, namely: my partner only gave me the card for care partners: 1/7 (14%)
How long did you use HiG on average at home? (%)	• 0–15 min: 2/10 (20%) • 16–30 min: 6/10 (60%) • 31–45 min: 0/0 (0%) • 46–60 min: 0/0 (0%) • Longer than 60 min: 2/10 (20%) • Other, namely: 0/0 (0%) • I do not remember: 0/0 (0%) • I did not use HiG: 0/0 (0%)	• 0–15 min: 2/7 (29%) • 16–30 min: 3/7 (43%) • 31–45 min: 0/0 (0%) • 46–60 min: 1/7 (14%) • Longer than 60 min: 0/0 (0%) • Other, namely: 0/0 (0%) • I do not remember: 0/0 (0%) • I did not use HiG: 1/7 (14%)
How often did you use HiG on average at home? (%)	• Once: 6/10 (60%) • Twice: 2/10 (20%) • Thrice: 1/10 (10%) • Four times: 0/0 (0%) • Five times: 1/10 (10%) • Other, namely: 0/0 (0%) • I do not remember: 0/0 (0%) • I did not use HiG: 0/0 (0%)	• Once: 4/7 (57%) • Twice: 0/0 (0%) • Thrice: 1/7 (14%) • Four times: 0/0 (0%) • Five times: 0/0 (0%) • Other, namely: eight times: 1/7 (14%) • I do not remember: 0/0 (0%) • I did not use HiG: 1/7 (14%)
Did you see the HiG poster? (%)**	• Yes: 2/10 (20%) • No: 7/10 (70%) • I do not remember: 0/0 (0%)	• Yes: 1/7 (14%) • No: 5/7 (71%) • I do not remember: 1/7 (14%)
Did you see the HiG video? (%)***	• Yes: 2/10 (20%) • No: 2/10 (20%) • I do not remember: 0/0 (0%)	• Yes: 2/7 (29%) • No: 5/7 (71%) • I do not remember: 0/0 (0%)
To what extent do you think HiG has helped you to discuss topics during the consultation? (%)	• not at all: 2/10 (20%) • a little: 5/10 (50%) • a lot: 3/10 (30%) • very much: 0/0 (0%) • I do not remember: 0/0 (0%)	• not at all: 1/7 (14%) • a little: 3/7 (43%) • a lot: 2/7 (29%) • very much: 1/7 (14%) • I do not remember: 0/0 (0%)
To what extent do you think HiG was easy to use? (%)	• not at all: 0/0 (0%) • a little: 2/10 (20%) • a lot: 4/10 (40%) • very much: 3/10 (30%) • I do not remember: 1/10 (10%)	• not at all: 0/0 (0%) • a little: 2/7 (29%) • a lot: 2/7 (29%) • very much: 3/7 (43%) • I do not remember: 0/0 (0%)
To what extent do you think your clinician got to know you as a person? (%)	• not at all: 1/10 (10%) • a little: 4/10 (40%) • a lot: 2/10 (20%) • very much: 3/10 (30%) • I do not remember: 0/0 (0%)	• not at all: 0/0 (0%) • a little: 2/7 (29%) • a lot: 5/7 (71%) • very much: 0/0 (0%) • I do not remember: 0/0 (0%)
To what extent do you plan to use HiG in consultations at the memory clinic in the future? (%)	• very unlikely: 1/10 (10%) • somewhat unlikely: 1/10 (10%) • neither: 2/10 (20%) • somewhat likely: 3/10 (30%) • very likely: 3/10 (30%)	• very unlikely: 0/0 (0%) • somewhat unlikely: 1/7 (14%) • neither: 0/0 (0%) • somewhat likely: 6/7 (86%) • very likely: 0/0 (0%)
To what extent would you like to recommend HiG to another person with cognitive complaints? (mean ± SD)	7.9 ± 1.6 (min = 5; max = 10)****	6 ± 2.9 (min = 0; max = 9)*****

Notes. *N = 2 people used HiG at home but did not select cards at home **N/A because this consult was in someone's home. ***N/A because we did not have an email address to send the video.****See Fig. 2 for the exact division. *****See Fig. 2b. for the exact division.

Additional in-depth views regarding the use of the tool emerged during the interviews. For instance, some people specifically indicated that they appreciated the opportunity to discuss the cards together with someone else at home, as this prompted them to share certain topics with their loved ones that they had not previously addressed explicitly. This is illustrated in the following quote:

‘*Well, because I noticed that, in particular, that card with family and friends, that moved me. I would never have talked about that. So it's good to have a card like that. Yes, you have to start by figuring it out, but then it's also important to talk about it at some point.’* (P_3, person with cognitive complaints).

In addition, several people with cognitive complaints mentioned that the cards facilitated the initiation of topics during the consultation:

*‘Well, at first I thought, you know, if you take a look at the first titles on those things, I would say no, but as it turned out, it has been useful after all. And I hadn't expected that beforehand.’* (P_4, person with cognitive complaints).

However, some also noted occasional uncertainty regarding what to fill in on the reverse side of the cards or not perceiving usefulness since they did not consider any of the topics to be of relevance:

*‘I didn't actually pick a card, because I didn't have a problem or anything.’* (P_6, person with cognitive complaints).

People with cognitive complaints did often not consciously notice the ‘Helder in Gesprek’ poster and video.

#### 3.5.2 Care partners

Care partners expressed a similar attitude towards ‘Helder in Gesprek’ in the evaluation questionnaire (see for detailed results Table 5). The majority of care partners indicated that the clinician got to know them and the person with cognitive complaints (if present) as a person (5/7) and said that they are ‘somewhat likely’ to use ‘Helder in Gesprek’ again in another consultation (6/7). Most, yet not all, care partners would recommend ‘Helder in Gesprek’ to others (6 ± 2.9; min = 0; max = 9; for frequencies see Table 5 and Fig. 2b). The following quotes exemplify the view of care partners, as provided in answer to an open-ended question:

*‘It helps me to find out how my husband feels about it, and above all, what is bothering him.’* (N_5, care partner).

*‘Great tool to add focus’* (N_9, care partner).

‘*Our questions would have been addressed also without them* [the cards].*’* (N_9, care partner).

‘*Many questions have been asked. The cards have helped me think about what I wanted to talk about.’* (N_1, care partner).

Care partners did often not consciously notice the ‘Helder in Gesprek’ poster and video.

### 3.6 Clinicians

Table 6 displays detailed results regarding clinicians' answers to the closed-ended questions that were part of the evaluation interview. Clinicians indicated that ‘Helder in Gesprek’ had helped them to get to know individual people with cognitive complaints or care partners better (5/11) or a lot better (3/11). They also positively evaluated the usability of ‘Helder in Gesprek’ (a little: 1/7; a lot: 4/7; very much: 2/7). In addition, most clinicians thought that ‘Helder in Gesprek’ positively affected the involvement of people with cognitive complaints and care partners during the consultation (a little: 1/7; a lot: 3/7; very much: 1/7), whereas a few did not necessarily think so (not at all: 2/7), and added that they considered the people visiting their memory clinic as already quite assertive. Results were mixed regarding clinicians' opinion whether ‘Helder in Gesprek’ affected their workflow (not at all: 2/7; a little: 3/7; a lot: 1/7; very much: 1/7): some indicated that discussing ‘Helder in Gesprek’ took extra time, whereas others mentioned that discussing the cards did not take extra time compared to the usual question of ‘how are you doing?’. These logistics and time considerations explain why some clinicians planned to use ‘Helder in Gesprek’ in the future (very likely: 1/7; somewhat likely: 2/7; neither: 1/7; somewhat unlikely: 3/7) and/or would recommend the tool to colleagues whereas others would not (6.6 ± 2.9; min = 2; max = 10; see Fig. 2c) or people with cognitive complaints (5.43 ± 2.4; min = 2; max = 8; see Fig. 2d).

**Table 6:** Evaluation of ‘Helder in Gesprek’ by clinicians (*n* = 7).

Did you see the HiG poster? (%)*	• Yes: 5/7 (71%) • No: 1/7 (14%) • I do not remember: 1/7 (14%)
**Did you see the HiG video for clinicians? (%)**	• Yes: 7/7 (100%) • No: 0/0 (0%) • I do not remember: 0/0 (0%)
**Did you see the HiG placemat?**	• Yes: 7/7 (100%) • No: 0/0 (0%) • I do not remember: 0/0 (0%)
**Did you use HiG during your consultations?**	• Yes: 7/7 (100%) • No: 0/0 (0%) • I do not remember: 0/0 (0%)
To what extent do you think HiG has helped you to get to know this specific person with cognitive complaints or care partner better? (%)**	• Not at all better: 0/0 (0%) • not better: 1/11 (9%) • neutral: 2/11 (18%) • better: 5/11 (46%) • a lot better: 3/11 (27%)
**To what extent do you think HiG was easy to use? (%)**	• not at all: 0/0 (0%) • a little: 1/7 (14%) • a lot: 4/7 (57%) • very much: 2/7 (29%)
**To what extent do you think HiG has affected your workflow? (%)**	• not at all: 2/7 (29%) • a little: 3/7 (43%) • a lot: 1/7 (14%) • very much: 1/7 (14%)
**To what extent do you think HiG has affected the involvement of people with cognitive complaints (and care partners) during the consultation? (%)**	• not at all: 2/7 (29%) • a little: 1/7 (14%) • a lot: 3/7 (43%) • very much: 1/7 (14%)
**To what extent do you plan to use HiG in consultations in the future? (%)**	• very likely: 1/7 (14%) • somewhat likely: 2/7 (29%) • neither: 1/7 (14%) • somewhat unlikely: 3/7 (43%) • very unlikely: 0/0 (0%)
**How likely is it that you will recommend HiG to a colleague? (mean** **± SD)**	6.6 ± 2.9 (min = 2; max = 10)
**How likely is it that you will recommend HiG to a person with cognitive complaints? (mean** **± SD)**	5.43 ± 2.4 (min = 2; max = 8)

Notes. *HiG= ‘Helder in Gesprek’. **asked after every individual consultation in which HiG was tested to people with cognitive complaints (n = 10) and care partners (n = 2) who had a one-on-one consult with a clinician. N = 1 missing due to data loss.

Qualitative analysis of clinicians' answers to the open-ended interview questions resulted in the following insights (for coding tree, see appendix 7). First, on the one hand, positive remarks were made regarding the usability and usefulness of ‘Helder in Gesprek’ that included: the tool being practical and concrete, visibly seeing the cards on the placemat seemed to support people with cognitive complaints with communicating about these topics, the tool required little work during the consultation, and seemed to add focus to the consultation. Moreover, one clinician explicitly noted their preference for the tool being a tangible, physical instrument that could be placed on the table, rather than a digital version displayed on a computer, as they felt that the latter could interfere with or diminish the quality of the patient–clinician relationship.

‘*Yes, I think it has a positive effect. I think people are a bit more focused. Like, some people make a list of all the things they don't want to forget. I think that's how it works. And also for the care partners. So you noticed that in one or two of those conversations, for example, that the care partners came back to the cards a bit more. So I think that, um… it does guide them. I think it's a kind of anchor.’* (ZV_8, clinician, neurologist).

Second, on the other hand, doubts about usefulness were expressed and regarded: the number of cards which was considered too much, the need for people with cognitive complaints to prepare at home, and the cards not being relevant to everyone. For instance, the tool was considered not suitable for people who deny their cognitive problems and are reluctant to visit the memory clinic, people with frontotemporal dementia, or people in advanced stages of dementia.

Third, the relevance of ‘Helder in Gesprek’ was explicitly acknowledged by some clinicians. About half of the clinicians agreed during the interviews that ‘Helder in Gesprek’ helped to discuss topics that would not have been discussed otherwise, helped to get to know people with cognitive complaints, and may have helped to facilitate the communication, especially during ‘difficult’ consultations. Others indicated that ‘Helder in Gesprek’ does not necessarily help to discuss ‘new’ topics or does not necessarily help to deepen the conversation [7].

*‘Yes, more than I had expected at first. So that surprised me. So that's quite something, I think. Um, because I felt like I knew this patient pretty well, and I was surprised by the cards the patient came up with. She also told an emotional story from the past, which was really surprising to me, because I didn't realize she was still dealing with that. Um, and there was another card, I can't remember the exact card off the top of my head, but it was also that I thought, oh, I expected different cards from you. So I found that surprising.’* (ZV_10, clinician, nurse).

‘*Oh, yes, I had an example of a care partner with whom I found the conversation quite difficult. She stuttered a lot and couldn't get her words out properly. And then she laid the card on the table anxiously, or rather, the card that showed she was worried about the future, and then she loosened up a bit, and I thought, oh yes, I actually really liked noticing that at the time.’* (ZV_1, clinician, nurse).

‘*Yes, but also that, compared to our current consultation practice, I don't yet see the added value… Yes, it may only be a few percent, but to say that this really makes a difference or that I have never done such a consultation before, I really think that I will become more open, which will lead to different conversations, I don't think so. And I think, based on my current estimate in terms of time, that the few percent you will gain is not proportional. And let alone in terms of cost-effectiveness and paper cards regarding the environment.’* (ZV_7, clinician, nurse).

Fourth, regarding implementation, clinicians suggested to not hand out ‘Helder in Gesprek’ to all people with cognitive complaints, but to offer it during follow-up consultations instead. The reason for this suggestion being that the clinician then knows the person better and is better able to estimate who would benefit from the tool and who might not. Clinicians generally also indicated that the logistics surrounding implementation of ‘Helder in Gesprek’ are quite burdensome, i.e. calling people who are scheduled for a consultation and sending the packages.

‘*Clinician: But if you look at the time frame for using ‘Helder in Gesprek’, well, yes, that is very complex. The investment required to call patients in advance, to explain things to them, whether in the context of the study or outside of it, and that turns out to be very difficult. So, yes, it's a bit of a double-edged sword. I think it's very applicable in the consultation room, but the work involved…*

*Interviewer: Hmm, yes, so the logistics are too much.*

*Clinician: Yes, the logistics and the phone calls, yes, and we are a very small team and… as clinicians, we are on our own and we don't have any support staff for that, and it's going to be very difficult to fit in. And I foresee that as an obstacle in any future use, especially in relation to what it will deliver.*’ (ZV_7, clinician, nurse).

Clinicians did often notice the poster, video, and ‘Helder in Gesprek’ placemat.

## 4 Discussion and conclusion

### 4.1 Discussion

The tool ‘Helder in Gesprek’ (HiG; translation ‘Clear in Conversation’) aims to enhance person-centred communication by supporting people with cognitive complaints to select and articulate topics they wish to discuss during consultations with their clinician [19]. The aim of this study was to assess the feasibility of ‘Helder in Gesprek’ in various Dutch real-world outpatient care settings among people with cognitive complaints, care partners, and clinicians. We showed that ‘Helder in Gesprek’ was overall well received, acceptable, and usable for people with cognitive complaints, their care partners, and clinicians. Most people with cognitive complaints indicated that ‘Helder in Gesprek’ helped them to discuss topics relevant to them with their clinician, felt that the clinician got to know them as a person during the consultation, planned to use this tool for future consultations, and would recommend the tool to others. Clinicians indicated that ‘Helder in Gesprek’ helped them to get to know individual people with cognitive complaints or their care partners better and most perceived a positive impact on their involvement in the consultation. Mixed results were found among clinicians as to whether ‘Helder in Gesprek’ (negatively) affected their workflow.

In total, fourteen out of twenty-one ‘Helder in Gesprek’ topic cards were discussed during the consultations, highlighting the wide variety of topics that people wanted to discuss with their clinician. Topics that currently receive limited attention during consultations in outpatient care settings, yet that were (often) chosen in our current study included the themes ‘needs’ and ‘experiences’, for example: *‘I would like to tell my clinician that I have experienced something traumatic in my life’* [15]. Similarly, cards addressing (previous and/or volunteer) work were picked by a (substantial) proportion of our sample to discuss with the clinician and usually related to what people miss about their old jobs or what their current job gives them, for example, feeling useful and social connections. This suggests that even when activity-related cards are chosen, the underlying need seems to be to discuss what they value in life. All four cards comprising the theme social environment were never chosen in the current study, whereas a previous study among people with cognitive complaints did find people wanting to discuss aspects of the social environment [13]. Given the small sample of the current study and the evidence-based development of the cards [19], future large-scale studies are needed to investigate how often each card is selected and discussed, and whether social environment-related cards can be left out or not. Revision of the specific texts on the cards might also be an avenue to explore.

Some clinicians indicated that ‘Helder in Gesprek’ had helped them to discuss topics that were important to the person with cognitive complaints as well as relevant to themselves, whereas a few others disagreed. Regarding the latter, one might contend that actively listening to what an individual considers important to discuss with their clinician could already be an important step in providing care for people with cognitive complaints, making them feel seen, acknowledged, and understood. Moreover, from their perspective, and resulting discussions, engaging in conversations about what matters to people with cognitive complaints in itself may yield positive effects, such as improved satisfaction, information recall, trust, dignity, (self-) respect, and overall wellbeing [9], [10], [11], [12], [13], [19]. Consequently, explicitly providing space for and responding to people's affective needs and experiences can be considered equally important as addressing their instrumental, informational, or pragmatic needs, all adding to the quality of care.

In the current study, the overall usability of ‘Helder in Gesprek’ was perceived as ‘good’ by people with cognitive complaints and care partners. Yet, concerns regarding the tool's complexity were also voiced, especially in the interviews. Especially the card sorting was considered too difficult for some people regarding the number of cards and given the cognitive load this task requires, specifically for people with dementia. Unfortunately, our sample size was too small to make any comparisons based on diagnosis. Nevertheless, re-design and options for further simplification should be explored to allow for an even more inclusive design. A large-scale study is recommended to get insight into which cards are frequently picked per setting and should thus stay in the card deck and which cards can be removed or merged with other cards. We also recommend to explore opportunities for a more inclusive re-design of the digital prototype of ‘Helder in Gesprek’ [19] with automated (AI) support for card sorting to reduce cognitive load.

This study suggests that the concept of ‘Helder in Gesprek’ is feasible and that the tool can support person-centred communication in the memory clinic. The following elements can be identified as the likely effective components of the tool. First, visibly seeing the cards on the placemat in the consultation room, appeared to support people with cognitive complaints with communicating about these topics and helped both people with cognitive complaints and clinicians to add focus during the consultation. One clinician commented that a digital version might disrupt or decrease the patient-clinician relationship. This reasoning is in line with previous research that states that using digital tools or computers during consultations may adversely affect the patient-clinician relationship and communication [39], [40], [41], [42], [43].

Second, the preparation at home seemed to be of value. Evaluation data shows that most people took on average 16–30 min to prepare. This time for reflection is not available during the consultation. During the development of ‘Helder in Gesprek’, we found that using this tool in the waiting room is not an option, as people may have limited mental capacity to think about topics that they want to discuss when waiting for the consult to start, for instance, due to nerves or external stimuli [19]. Some clinicians, however, viewed the preparation at home as a potential burden for people with cognitive complaints and therefore as a barrier for implementation of the tool. In the current study, two people forgot to bring the cards that they picked at home but were able to retrieve these cards when offered in the consultation room.

Third, the tool can facilitate person-centred care because it explicitly puts the ‘power’ of what will be discussed and is put on the agenda in the hands of people with cognitive complaints. People may decide for themselves what they do and do not necessarily want to discuss with the clinician, adding to their autonomy and sense of empowerment. Additionally, ‘Helder in Gesprek’ may provide an easy way to address topics that may be harder to talk about or initiate for some people in a way that upholds their dignity, whilst also opening the space for discussion and support. However, this study shows that people with cognitive complaints seem to need an explicit invitation by the clinician at the beginning of the consultation to do so. This finding aligns with previous research suggesting that people with cognitive complaints do not often initiate topics themselves, and that clinicians typically govern the flow of information during consultations [15], [16], [18], [44]. Finally, the majority of people with cognitive complaints used ‘Helder in Gesprek’ at home together with a care partner. This aspect was valued by several people with cognitive complaints as well as care partners, as this facilitated conversations about what mattered to the person with cognitive complaints and enabled care partners to gain insight into someone's values, emotions, preferences, and/or priorities.

People with cognitive complaints indicated in the development as well as in the current study that they want their clinician to get to know them as a person as early as possible in the care trajectory [19]. However, in the current study, clinicians suggested that ‘Helder in Gesprek’ would be more appropriate for follow-up consultations or in case manager settings. A reason is that clinicians consider themselves better able to respond to or address follow-up actions once a diagnosis is made. However, one could argue that merely listening to and acknowledging what the person with cognitive complaints wants to share about themselves is important and sufficient and does not necessarily require a definitive diagnosis, nor concrete actions or advise. Moreover, this information could inform the diagnostic process, as it will help to clarify what the person with complaints is actually seeking for; currently, this is often not discussed at the start of the diagnostic pathway [15].

The ‘Helder in Gesprek’ tool aims to improve person-centred communication in memory clinics, geriatrics departments, and case manager settings. In other clinical fields, topic cards have also been designed and developed that aim to improve patient-clinician communication. For instance, conversation cards have been developed and evaluated as tools to support collaborative agenda setting between people with type 2 diabetes and clinicians [45]. Early-stage evaluations indicated positive responses from patients and nurses, more patient-centred communication, and good levels of acceptability and feasibility [45]. In a subsequent effect study, patients reported that the conversation cards were helpful in raising issues they wished to discuss with their clinicians, and the authors suggested that the cards might broaden the scope of diabetes consultations and help patients remember their questions [46]. These findings are consistent with the results of our feasibility study. Similarly, cards displaying pictures or statements have been shown to support the expression of thoughts and feelings in conversations with people living with obesity, as well as with healthy adults and patients in palliative care [47], [48], [49], [50]. In palliative care settings, the use of such cards was considered feasible and beneficial and was appreciated by patients [50]. Another study in hospital settings demonstrated that patients selected a wide range of topics using the Tell-us-Cards, with most chosen topics focusing on psychosocial needs [51], This aligns with our findings, in which the most frequently selected cards related to ‘needs’ or ‘experiences’. Results from the Tell-us Card study further showed that most patients considered the cards beneficial, while clinicians reported barriers to implementing them in daily practice [51], which is also reflected in our findings.

Taken together, the results of this study demonstrate the importance of explicitly inviting people with cognitive complaints to share what they find important to discuss. For instance, using questions such as ‘*What is important for you to discuss today?*’, ‘*What do you find important that I know about your life and situation?*’, and/or questions specifically tailored towards the themes needs, experiences, and activities, such as ‘*What kind of activities give you energy or fulfilment?’*, ‘*To what extent do you worry about your situation at the moment or in the future?*’, or ‘*How does this current situation in your life make you feel?*’ This study also underscores the potential benefit of allowing people with cognitive complaints to take initiative and guide the flow of the consultation instead of a fixed agenda solely provided by the clinician. In addition, ‘Helder in Gesprek’ may not only support people with cognitive complaints, care partners, and clinicians, but may also be used as an educational tool in medical education. The tool may help students to practice their communication skills, including the ability to adapt to a specific person.

#### 4.1.1 Limitations

Some study limitations need to be considered. First, since we explained the purpose of the study during recruitment, participants with an interest in improving communication may have been more likely to participate. Additionally, we were only able to recruit one neurologist and one case manager (compared to multiple (specialized) nurses). This may suggest that the topic of person-centred communication is more appealing to nurses. These selection biases negatively affect the validity of study findings, e.g., in the sense that we might not have collected more critical views on the feasibility of ‘Helder in Gesprek’. Second, we have a limited number of participants, minimizing the external validity of our study. Despite multiple visits by the researcher, personal explanations, and friendly reminders, recruitment appeared to be challenging due to limited time, other studies that were prioritized, staff shortages and holidays. We were unfortunately only able to recruit one participant in a case manager setting, which limits insight into the feasibility of ‘Helder in Gesprek’ in this particular setting. Third, results from qualitative analyses of the consultations must be interpreted with caution, because we did not compare the ‘Helder in Gesprek’ consultations with regular consultations. Moreover, we did not formally assess inter-coder reliability during thematic analysis, which limits the reliability of the findings. However, codes were checked by a second researcher and discussed until consensus was reached and themes were discussed among all co-authors. Fourth, by offering people with cognitive complaints and care partners the opportunity to fill out the evaluation questionnaire at home, recall bias may have been introduced. However, we considered it important to accommodate to their preferences and wanted them to take their time to complete the questionnaire, which might have improved reliability.

#### 4.1.2 Future research

Future research is needed to confirm the tool's effectiveness and efficiency, and explore whether ‘Helder in Gesprek’ improves outcomes and experiences. Such research requires a larger study sample and a randomized-controlled study design. Outcomes relevant to consider for such a study may regard three levels: 1) the level of the person with cognitive complaints and care partners (e.g. including the degree of involvement and empowerment, and patient-reported experiences measures (PREMs)), 2) the level of the clinician (e.g. including outcomes such as self-efficacy in communication and whether the tool helps clinicians to get to know the people with cognitive complaints better), and 3) the level of the care process (e.g., whether the tool helps to (in-)directly shorten care trajectories or whether the tool may help to improve the information flow between different care settings, such as a memory clinic, case manager, general practitioner, or psychologist). Moreover, a trial should take (cultural) diversity into account. Subgroup analyses are also recommended to explore whether ‘Helder in Gesprek’ may be more suitable and usable for certain people with cognitive complaints, such as people with SCD, MCI, or dementia. Other directions for future research comprise co-design sessions among culturally diverse populations for validation and refinement of the tool and exploration of whether there is a need for a digital version. Moreover, future research could explore the suitability and options for implementation in non-dementia related care settings, as the topics on the cards are likely to be relevant to other medical fields as well. Finally, implementation research is needed to further guide the application of the ‘Helder in Gesprek’ and person-centred communication in clinical practice.

### 4.2 Innovation

We developed and tested a new tool to support person-centred communication in the outpatient care setting specifically for people with cognitive complaints. To the best of our knowledge, this tool is a first of its kind in memory clinics, geriatrics departments, and case manager settings. The tool combines textual and visual information, making it highly usable for people who may have trouble to express themselves during consultations. Also, the tool explicitly provides people with cognitive complaints with power for agenda setting and agency over what they do and do not want to share with the clinician. The latter aspect can be seen as an improvement in healthcare (communication) in general, moving towards a person-centred approach, yet the real innovative value of ‘Helder in Gesprek’ lies specifically in its focus on people with cognitive complaints. People with cognitive complaints or impairments may face stigma and are often viewed as a marginalised group in society, with limited agency and knowledge [52], [53], [54], [55], [56]. Yet they are knowledgeable in important ways, for example about their own experiences of cognitive complaints and how they make sense of those experiences [57]. If this knowledge is not recognised and valued, epistemic injustice may occur [58], [59]. As a result, people with cognitive complaints may find it more difficult to be fully and equally involved in consultations and to share their story. This imbalance can be seen in patient-clinician communication, for example when clinicians set the consultation agenda on their own, or when people with cognitive complaints do not feel the space for, and/or able to, tell their story or have difficulty expressing themselves and their experiences. Tools, such as ‘Helder in Gesprek’, may help to minimize this form of epistemic injustice and facilitate person-centred communication by offering people with cognitive complaints support and power. Next to that, the tool also supports clinicians to create space in consultations, to actively encourage people with cognitive complaints to share their experiences in ways that suit them and, consequently, provide them with care that fits who they are as a person.

### 4.3 Conclusion

This study shows that the concept of the tool ‘Helder in Gesprek’ is acceptable for improving person-centred care and is considered usable among people with cognitive complaints, care partners, and clinicians. The majority of people with cognitive complaints and care partners indicate that ‘Helder in Gesprek’ helped them to discuss relevant topics during the consultation and that they felt seen as a person by the clinician. Similarly, the majority of clinicians indicate that ‘Helder in Gesprek’ helped them to get to know the person with cognitive complaints or care partner better. Based on this study, the tool may be regarded as feasible, provided that the barriers identified herein are addressed. Future research is needed to facilitate implementation and confirm the effectiveness and sustainability in clinical practice.

## AI statement

During the preparation of this manuscript, the authors used DeepL Write and Perplexity to assist with grammar checking and language improvement purposes only. The authors reviewed and edited all AI-generated grammar and language suggestions and take full responsibility for the final version of the manuscript.

## CRediT authorship contribution statement

**Tanja J. de Rijke:** Writing – original draft, Visualization, Project administration, Methodology, Investigation, Formal analysis, Data curation, Conceptualization. **Thomas Engelsma:** Writing – review & editing, Supervision, Formal analysis, Conceptualization. **Heleen M.A. Hendriksen:** Writing – review & editing, Investigation. **Dianne Vasseur:** Writing – review & editing. **Huiberdina L. Koek:** Writing – review & editing. **Joost W. van den Berg:** Writing – review & editing. **Niki Schoonenboom:** Writing – review & editing. **Ellen M.A. Smets:** Writing – review & editing, Supervision, Funding acquisition, Conceptualization. **Leonie N.C. Visser:** Writing – review & editing, Supervision, Funding acquisition, Conceptualization.

## Consent to participate

Participants provided written informed consent prior to participation to conduct and publish the study.

## Ethical considerations

The Medical Ethics Committee of the Amsterdam UMC, location AMC, approved the study (METC number P1a 2025.0513). This study is conducted in line with the Declaration of Helsinki.

## Funding

ES and LV are recipients of ABOARD, which is a public–private partnership receiving funding from 10.13039/501100001826ZonMW (#73305095007) and Health∼Holland, Topsector Life Sciences & Health (PPP-allowance; #LSHM20106). More than 30 partners participate in ABOARD. ABOARD also receives funding from Edwin Bouw Fonds and Gieskes-Strijbisfonds. www.aboard-project.nl. ES is a recipient of TAP-dementia, a ZonMw funded project (#10510032120003) in the context of the Dutch National Dementia Strategy. The sponsors had no involvement in the study design; in the collection, analysis, and interpretation of data; in the writing of the report; or in the decision to submit the manuscript for publication.

## Declaration of competing interest

The research of LV has been funded by ZonMW, Alzheimer Nederland, Health∼Holland, Topsector Life Sciences & Health, EISAI and Amsterdam public health research institute. TR, TE, HH, DV, DK, JB, NS, and ES report no competing interests.

## Data Availability

The data that support the findings of this study are not openly available due to reasons of study participant privacy. Data are in controlled data access storage at the Amsterdam UMC, location AMC.

## References

[1] Nichols E., Steinmetz J.D., Vollset S.E., Fukutaki K., Chalek J., Abd-Allah F. (2022). Estimation of the global prevalence of dementia in 2019 and forecasted prevalence in 2050: an analysis for the global burden of disease study 2019. Lancet Public Health.

[2] Beard J.R., Bloom D.E. (2015). Towards a comprehensive public health response to population ageing. Lancet.

[3] Davis B. (2005).

[4] Ryan E.B., Meredith S.D., MacLean M.J., Orange J.B. (1995). Changing the way we talk with elders: promoting health using the communication enhancement model. Int J Aging Hum Dev.

[5] Savundranayagam M.Y., Moore-Nielsen K. (2015). Language-based communication strategies that support person-centered communication with persons with dementia. Int Psychogeriatr.

[6] O’Rourke D.J., Lobchuk M.M., Thompson G.N., Lengyel C. (2022). Expanding the conversation: a person-centred communication enhancement model. Dementia.

[7] Hafskjold L., Sundler A.J., Holmström I.K., Sundling V., van Dulmen S., Eide H. (2015). A cross-sectional study on person-centred communication in the care of older people: the COMHOME study protocol. BMJ Open.

[8] Constand M.K., MacDermid J.C., Dal Bello-Haas V., Law M. (2014). Scoping review of patient-centered care approaches in healthcare. BMC Health Serv Res.

[9] Batbaatar E., Dorjdagva J., Luvsannyam A., Savino M.M., Amenta P. (2017). Determinants of patient satisfaction: a systematic review. Perspect Public Health.

[10] Monden K.R., Gentry L., Cox T.R. (2016).

[11] Kinmonth A.L., Woodcock A., Griffin S., Spiegal N., Campbell M.J. (1998). Randomised controlled trial of patient centred care of diabetes in general practice: impact on current wellbeing and future disease risk. The Diabetes Care From Diagnosis Research Team. BMJ.

[12] Fruijtier A.D., van der Schaar J., van Maurik I.S., Zwan M.D., Scheltens P., Bouwman F. (2022). Identifying best practices for disclosure of amyloid imaging results: a randomized controlled trial. Alzheimers Dement.

[13] de Rijke T.J., Hendriksen H.M., Fruijtier A.D., van Harten A.C., van Leeuwenstijn-Koopman M.S., van de Giessen E.M. (2025). Needs expressed by people with subjective cognitive decline during amyloid PET disclosure consultations: an observational study. Patient Educ Couns.

[14] Kitwood T., Bredin K. (1992). Towards a theory of dementia care: personhood and well-being. Ageing Soc.

[15] Visser L.N., Fruijtier A., Kunneman M., Bouwman F.H., Schoonenboom N., Staekenborg S.S. (2023). Motivations of patients and their care partners for visiting a memory clinic. A qualitative study. Patient Educ Couns.

[16] Visser L.N., Kunneman M., Murugesu L., van Maurik I., Zwan M., Bouwman F.H. (2019). Clinician-patient communication during the diagnostic workup: the ABIDE project. Alzheimers Dement Diagn Assess Dis Monit.

[17] Kunneman M., Pel-Littel R., Bouwman F.H., Gillissen F., Schoonenboom N.S., Claus J.J. (2017). Patients’ and caregivers’ views on conversations and shared decision making in diagnostic testing for Alzheimer’s disease: the ABIDE project. Alzheimer’s & Dementia (New York, N Y).

[18] Fruijtier A.D., Visser L.N., Bouwman F.H., Lutz R., Schoonenboom N., Kalisvaart K. (2020). What patients want to know, and what we actually tell them: the ABIDE project. Alzheimers Dement Transl Res Clin Interv.

[19] de Rijke T.J., Kaijser K.K., Vasseur D., Tasköprü H., Huisman L., van Gils A.M. (2026). Design and development of ‘Helder in Gesprek’: a tool to support person-centred communication in memory clinics. Digit Health.

[20] de Rijke T.J., Kaijser K.K., Vasseur D., Smets E.M., Visser L.N., Derksen M. (2025). Usability assessment of a digital tool to enhance person–clinician communication in the memory clinic: an expert evaluation. Digit Health.

[21] Bowen D.J., Kreuter M., Spring B., Cofta-Woerpel L., Linnan L., Weiner D. (2009). How we design feasibility studies. Am J Prev Med.

[22] Gruters A.A.A., Ramakers I., Kessels R.P.C., Bouwman F.H., Olde Rikkert M.G.M., Blom M.M. (2019). Development of memory clinics in the Netherlands over the last 20 years. Int J Geriatr Psychiatry.

[23] Jack C.R., Bennett D.A., Blennow K., Carrillo M.C., Dunn B., Haeberlein S.B. (2018). NIA-AA research framework: toward a biological definition of Alzheimer’s disease. Alzheimers Dement.

[24] Chen Z., Yu J., Song Y., Chui D. (2010). Aging Beijing: challenges and strategies of health care for the elderly. Ageing Res Rev.

[25] Association aAs (2026).

[26] Medicine EFoI. Geriatrics. n.d.

[27] Dreves M.A., van Harten A.C., Visser L.N., Rhodius-Meester H., Köhler S., Kooistra M. (2023). Rationale and design of the ABOARD project (a personalized medicine approach for Alzheimer’s disease). Alzheimers Dement Transl Res Clin Interv.

[28] Association WM (2013). World medical association declaration of Helsinki: ethical principles for medical research involving human subjects. JAMA.

[29] Philips (2026).

[30] Given L.M. (2008).

[31] Corp. I (2021).

[32] software V (2022).

[33] Mayring P. (2015).

[34] Miles M.B., Huberman A.M., Saldaña J. (2018).

[35] Booth A., Hannes K., Harden A., Noyes J., Harris J., Tong A. (2014). COREQ (consolidated criteria for reporting qualitative studies). Guidel Report Health Res.

[36] Tong A., Sainsbury P., Craig J. (2007). Consolidated criteria for reporting qualitative research (COREQ): a 32-item checklist for interviews and focus groups. Int J Qual Health Care.

[37] Pleijers A., De Vries R. (2021).

[38] Manalastas G., Noble L.M., Viney R., Griffin A.E. (2021). What does the structure of a medical consultation look like? A new method for visualising doctor-patient communication. Patient Educ Couns.

[39] Crampton N.H., Reis S., Shachak A. (2016). Computers in the clinical encounter: a scoping review and thematic analysis. J Am Med Inform Assoc.

[40] Greatbatch D., Heath C., Campion P., Luff P. (1995). How do desk-top computers affect the doctor-patient interaction. Fam Pract.

[41] Greatbatch D., Luff P., Heath C., Campion P. (1993). Interpersonal communication and human-computer interaction: an examination of the use of computers in medical consultations. Interact Comput.

[42] Booth N., Robinson P., Kohannejad J. (2004). Identification of high-quality consultation practice in primary care: the effects of computer use on doctor-patient rapport. Inform Prim Care.

[43] Margalit R.S., Roter D., Dunevant M.A., Larson S., Reis S. (2006). Electronic medical record use and physician–patient communication: an observational study of Israeli primary care encounters. Patient Educ Couns.

[44] Gobat N., Kinnersley P., Gregory J.W., Robling M. (2015). What is agenda setting in the clinical encounter? Consensus from literature review and expert consultation. Patient Educ Couns.

[45] Lomborg K., Munch L., Krøner F.H., Elwyn G. (2022). “less is more”: a design thinking approach to the development of the agenda-setting conversation cards for people with type 2 diabetes. PEC Innov.

[46] Munch L., Stensgaard S., Feinberg M.B., Elwyn G., Lomborg K. (2024). Evaluating the effect of conversation cards on agenda-setting in annual diabetes status visits: a multi-method study. Patient Educ Couns.

[47] Matteson C.L., Merth T.D., Finegood D.T. (2014). Health communication cards as a tool for behaviour change. ISRN Obes.

[48] Van Scoy L.J., Reading J.M., Scott A.M., Green M.J., Levi B.H. (2016). Conversation game effectively engages groups of individuals in discussions about death and dying. J Palliat Med.

[49] Lankarani-Fard A., Knapp H., Lorenz K.A., Golden J.F., Taylor A., Feld J.E. (2010). Feasibility of discussing end-of-life care goals with inpatients using a structured, conversational approach: the go wish card game. J Pain Symptom Manag.

[50] Rasmussen B.H., Fürst C.J., Malmström M., Beck I., Hagelin C.L., Pranter C. (2019). Using cards to facilitate conversations about wishes and priorities of patients in palliative care. J Hosp Palliat Nurs.

[51] van Belle E., Huisman-De Waal G., Vermeulen H., Heinen M. (2020). Feasibility and early effectiveness of the tell-us card communication tool to increase in-hospital patient participation: a cluster randomised controlled pilot study. Scand J Caring Sci.

[52] Read S.T., Toye C., Wynaden D. (2017). Experiences and expectations of living with dementia: a qualitative study. Collegian.

[53] Stockwell-Smith G., Moyle W., Kellett U. (2019). The impact of early-stage dementia on community-dwelling care recipient/carer dyads’ capacity to self-manage. J Clin Nurs.

[54] Evans-Lacko S., Bhatt J., Comas-Herrera A., D’Amico F., Farina N., Gaber S. (2019).

[55] Nguyen T., Li X. (2020). Understanding public-stigma and self-stigma in the context of dementia: a systematic review of the global literature. Dementia.

[56] Rewerska-Juśko M., Rejdak K. (2020). Social stigma of people with dementia. J Alzheimer’s Dis.

[57] Wylie A. (2013). Science and Other Cultures.

[58] Fricker M. (2007).

[59] Bhakuni H., Abimbola S. (2021). Epistemic injustice in academic global health. Lancet Glob Health.

