# Supplementary material for: A tool supporting person-centred communication among people with cognitive complaints, care partners, and clinicians: a feasibility study
Source: PEC Innov. 2026 Sep 10;9:100504. doi: 10.1016/j.pecinn.2026.100504 (PMC13599661; doi:10.1016/j.pecinn.2026.100504)
Supplement: Supplementary file 2 [file mmc1.docx]

**Appendix for manuscript ‘A tool supporting person-centred communication among people with cognitive complaints, care partners, and clinicians: a feasibility study’**

**Appendix 1. Overview of all topic cards in the ‘Helder in Gesprek’ tool**

*Activities*

- I would like to tell my clinician that I stay at home more than I used to.
- I would like to tell my clinician that (volunteer) work is important to me.
- I would like to tell my clinician that I enjoy meeting up with friends and/or family.
- I would like to tell my clinician that faith or spirituality plays a role in my life.
- I would like to tell my clinician that I am no longer employed.
- I would like to tell my clinician that I enjoy exercise.

*Social environment*

- I would like to tell my clinician that I feel a lot of support from the people around me.
- I would like to tell my clinician that people treat me differently than they used to.
- I would like to tell my clinician that I live with other people.
- I would like to tell my clinician that I am often on my own.

*Experiences*

- I would like to tell my clinician that I sometimes find it difficult to ask for help.
- I would like to tell my clinician that I have my own idea about what is going on with me.
- I would like to tell my clinician that I am reluctant to attend this appointment.
- I would like to tell my clinician that I feel good about myself.
- I would like to tell my clinician that I feel a certain emotion strongly.
- I would like to tell my clinician that I have experienced something traumatic in my life.

*Needs*

- I would like to tell my clinician that I am concerned about something.
- I would like to tell my clinician that I need advice or more information.
- I would like to tell my clinician that a lot is happening in my life.
- I would like to tell my clinician that I would like to prepare for the future.

*Other*

- There is another topic I would like to discuss, namely ...

*Care partner*

- As care partner, I find it important to tell the clinician that …

**Appendix 2. Observation guides**

1. **Is HiG used during the consultation?**
   - Yes
   - No
2. **Is HiG used ‘correctly’ during the consultation? If not, how?**
   - Yes
   - Not
3. **Who initiates the use of HiG during the consultation?**
   - Patient
   - Care partner
   - Clinician
4. **When is HiG discussed during the consultation?**
   - At the beginning
   - At the middle
   - At the end
5. **Which HiG cards are discussed during the consultation?**
   - I would like to tell my clinician that I feel a lot of support from the people around me.
   - I would like to tell my clinician that people treat me differently than they used to.
   - I would like to tell my clinician that I live with other people.
   - I would like to tell my clinician that I am often on my own.
   - I would like to tell my clinician that I sometimes find it difficult to ask for help.
   - I would like to tell my clinician that I have my own idea about what is wrong with me.
   - I would like to tell my clinician that I am reluctant to attend this appointment.
   - I would like to tell my clinician that I feel good about myself.
   - I would like to tell my clinician that I feel a certain emotion strongly.
   - I would like to tell my clinician that I have experienced something traumatic in my life.
   - I would like to tell my clinician that I stay at home more than I used to.
   - I would like to tell my clinician that (volunteer) work is important to me.
   - I would like to tell my clinician that I enjoy meeting up with friends and/or family.
   - I would like to tell my clinician that faith or spirituality plays a role in my life.
   - I would like to tell my clinician that I am no longer employed.
   - I would like to tell my clinician that I enjoy exercise.
   - I would like to tell my clinician that I am concerned about something.
   - I would like to tell my clinician that I need advice or more information.
   - I would like to tell my clinician that a lot is happening in my life.
   - I would like to tell my clinician that I would like to prepare for the future.
   - There is another topic I would like to discuss, namely ...
   - I don't remember
   - I did not pick any card
6. **Are the backside of the cards filled in?**
   - Yes
   - No
7. **How often is HiG discussed?**
8. **How long is HiG discussed?**
9. **How long is this consultation in total?**

**Appendix 3. Supported questionnaire for people with cognitive complaints**

1. **What is your age (in years)?**

………………………………………………………….

2. **What is your gender?**
   - Man
   - Woman
   - Non-binary person
   - Other, namely: …………………………..
3. **Which school or education did you last complete with a diploma?**
   - None or elementary school
   - Primary or preparatory vocational education (vso, vmbo-b, vmbo-k, vmbo-g, vmbo-t, mavo, mulo, lts, leao, lhno of meao)
   - Lower grades of secondary school of HAVO or VWO
   - Assistant training (mbo-1)
   - Higher grades of secondary school of HAVO, VWO, HBS, or MMS
   - Basic vocational training (mbo-2)
   - Vocational training (mbo-3)
   - Middle management and specialist education (mbo-4)
   - Higher vocational education (hbo, heao, or hts)
   - University
4. **In what country were you born?**

- The Netherlands
- Other, namely: ……………………………………………

5. **Which language or languages do you speak at home?**

- Dutch
- English
- Other, namely: ………………………………………………

6. **Did you use HiG at home?**
   *There are no right or wrong answers.*
   - Yes
   - No
   - I do not remember
7. **With whom did you use HiG at home prior to the consultation?**
   *There are no right or wrong answers.*
   - Alone
   - With my partner
   - With a family member (such as a brother, sister, niece or nephew)
   - With my child(ren)
   - With a friend
   - With an acquaintance
   - With someone else
   - I do not remember
   - I did not use HiG
8. **How long did you use HiG on average at home?**
   *Please make an estimation if you do not remember exactly. There are no right or wrong answers.*
   - 0-15 minutes
   - 16-30 minutes
   - 31-45 minutes
   - 46-60 minutes
   - Longer than an hour
   - Other, namely: ……………………………..
   - I do not remember
   - I did not use HiG
9. **How often did you use HiG at home?**
   *Please make an estimation if you do not remember exactly. There are no right or wrong answers.*
   - Once
   - Twice
   - Thrice
   - Four times
   - Five times
   - Other, namely: ………………………………
   - I do not remember
   - I did not use HiG
10. **Did you see the HiG poster?**
    *There are no right or wrong answers.*
    - Yes
    - No
    - I do not remember
11. **Did you see the HiG video?**
    *There are no right or wrong answers.*
    - Yes
    - No
    - I do not remember
12. **Which cards did you pick to discuss with the clinician?**
    *You can choose a maximum of three answers. There are no right or wrong answers.*
    - I would like to tell my clinician that I feel a lot of support from the people around me.
    - I would like to tell my clinician that people treat me differently than they used to.
    - I would like to tell my clinician that I live with other people.
    - I would like to tell my clinician that I am often on my own.
    - I would like to tell my clinician that I sometimes find it difficult to ask for help.
    - I would like to tell my clinician that I have my own idea about what is wrong with me.
    - I would like to tell my clinician that I am reluctant to attend this appointment.
    - I would like to tell my clinician that I feel good about myself.
    - I would like to tell my clinician that I feel a certain emotion strongly.
    - I would like to tell my clinician that I have experienced something traumatic in my life.
    - I would like to tell my clinician that I stay at home more than I used to.
    - I would like to tell my clinician that (volunteer) work is important to me.
    - I would like to tell my clinician that I enjoy meeting up with friends and/or family.
    - I would like to tell my clinician that faith or spirituality plays a role in my life.
    - I would like to tell my clinician that I am no longer employed.
    - I would like to tell my clinician that I enjoy exercise.
    - I would like to tell my clinician that I am concerned about something.
    - I would like to tell my clinician that I need advice or more information.
    - I would like to tell my clinician that a lot is happening in my life.
    - I would like to tell my clinician that I would like to prepare for the future.
    - There is another topic I would like to discuss, namely ...
    - I don't remember
    - I did not pick any card

**10. You have now reached the end of the questionnaire. Do you have any other comments or questions?**

**Appendix 4. Topic guide interview with cognitive complaints**

1. **Can you explain why you visited the memory clinic? What type of appointment was it** (e.g. initial consultation, results consultation, or follow-up appointment)?
2. **Did you look at the contents of the HiG box? Can you please elaborate?**
3. **Did you use HiG at home before your consultation with the clinician at the memory clinic? Can you please elaborate?**
4. **Did you use HiG during the consultation with your clinician? Can you please elaborate?**
5. **To what extent do you think HiG has helped you to discuss topics during the consultation?**
   - Not at all
   - A little
   - A lot
   - Very much
6. **Can you please elaborate?** [regarding question 5]
7. **To what extent do you think HiG was easy to use?**
   - Not at all
   - A little
   - A lot
   - Very much
8. **Can you please elaborate?** [regarding question 7]
9. **To what extent do you think your clinician got to know you as a person?**
   - Not at all
   - A little
   - A lot
   - Very much
10. **Can you please elaborate?** [regarding question 9]
11. **To what extent do you plan to use HiG in consultations at the memory clinic in the future? (%)**
    - Very likely
    - Somewhat likely
    - Neither
    - Somewhat unlikely
    - Very unlikely
12. **Can you please elaborate?** [regarding question 11]
13. **To what extent would you like to recommend HiG to another patient?** 0= not at all likely and 10= very likely

| 0 | 1 | 2 | 3 | 4 | 5 | 6 | 7 | 8 | 9 | 10 |
| --- | --- | --- | --- | --- | --- | --- | --- | --- | --- | --- |

14. **Can you please elaborate?** [regarding question 13]

**Appendix 5. Questionnaire for care partners**

1. **Your care partner had a consultation with a clinician at the memory clinic. What is your relationship to each other? I am the..**
   - Partner
   - Child
   - Family member
   - Friend
   - Acquaintance
   - Other, namely:
2. **What is your age (in years)?**

………………………………………………………….

3. **What is your gender?**
   - Man
   - Woman
   - Non-binary person
   - Other, namely: …………………………..
4. **Which school or education did you last complete with a diploma?**
   - None or elementary school
   - Primary or preparatory vocational education (vso, vmbo-b, vmbo-k, vmbo-g, vmbo-t, mavo, mulo, lts, leao, lhno of meao)
   - Lower grades of secondary school of HAVO or VWO
   - Assistant training (mbo-1)
   - Higher grades of secondary school of HAVO, VWO, HBS, or MMS
   - Basic vocational training (mbo-2)
   - Vocational training (mbo-3)
   - Middle management and specialist education (mbo-4)
   - Higher vocational education (hbo, heao, or hts)
   - University
5. **In what country were you born?**

- The Netherlands
- Other, namely: ……………………………………………

6. **Which language or languages do you speak at home?**

- Dutch
- English
- Other, namely: ………………………………………………

7. **Did you look at the content of the HiG box?**

*There are no right or wrong answers.*

- Yes
- No
- I do not remember

**Can you please elaborate?** [regarding question 7]

8. **Did you use HiG at home prior to the consultation in the memory clinic?**
   *There are no right or wrong answers.*
   - Yes
   - No
   - I do not remember

**Can you please elaborate?** [regarding question 7]

9. **If you used ‘Clear Communication’ at home before the conversation with the care provider, did you use it together with your loved one?** *There are no right or wrong answers.*
   - Yes, I used HiG together with my care partner.
   - No, I used HiG on my own
   - I used ‘Clear Communication’ together with my loved one and on my own
   - I did not use ‘Clear Communication’
   - Other, namely: …………………………………
10. **How long did you use HiG on average at home?**
    *Please make an estimation if you do not remember exactly. There are no right or wrong answers.*
    - 0-15 minutes
    - 16-30 minutes
    - 31-45 minutes
    - 46-60 minutes
    - Longer than an hour
    - Other, namely: ……………………………..
    - I do not remember
    - I did not use HiG
11. **How often did you use HiG at home?**
    *Please make an estimation if you do not remember exactly. There are no right or wrong answers.*
    - Once
    - Twice
    - Thrice
    - Four times
    - Five times
    - Other, namely: ………………………………
    - I do not remember
    - I did not use HiG
12. **Did you see the HiG poster?**
    *There are no right or wrong answers.*
    - Yes
    - No
    - I do not remember
13. **Did you see the HiG video?**
    *There are no right or wrong answers.*
    - Yes
    - No
    - I do not remember
14. **Did you use HiG during the consultation with the clinician?**
    - Yes
    - No
    - I do not remember

**Can you please elaborate?** [regarding question 14]

15. **Which topic(s) did you write on the card for care partners?**
16. **To what extent do you think HiG has helped you to discuss topics during the consultation?**
    - Not at all
    - A little
    - A lot
    - Very much

**Can you please elaborate?** [regarding question 16]

17. **To what extent do you think HiG was easy to use?**
    - Not at all
    - A little
    - A lot
    - Very much

**Can you please elaborate?** [regarding question 17]

18. **To what extent do you think your clinician got to know you as a person?**
    - Not at all
    - A little
    - A lot
    - Very much

**Can you please elaborate?** [regarding question 18]

19. **To what extent do you plan to use HiG in consultations at the memory clinic in the future?**
    - Very likely
    - Somewhat likely
    - Neither
    - Somewhat unlikely
    - Very unlikely

**Can you please elaborate?** [regarding question 19]

15. **To what extent would you like to recommend HiG to another care partner?** 0= not at all likely and 10= very likely

| 0 | 1 | 2 | 3 | 4 | 5 | 6 | 7 | 8 | 9 | 10 |
| --- | --- | --- | --- | --- | --- | --- | --- | --- | --- | --- |

**Can you please elaborate?** [regarding question 17]

16. **You have now reached the end of the questionnaire. Do you have any other comments or questions?**

**Appendix 6. Mini questionnaire for clinicians after every consultation**

**Date and time consultation:**

…………………………………………………………

1. **To what extent do you think HiG has helped you to get to know this specific person with cognitive complaints better? (%)**
   a. A lot better
   b. Better
   c. Neutral
   d. Not better
   e. Not at all better
2. **What did you find helpful/unhelpful about using HiG with this specific person with cognitive complaints?**

**Appendix 7. Topic guide interview clinicians**

1. **What is your gender?**
2. **What is your age?**
3. **What is your profession?**
   - Nurse
   - Specialized nurse
   - Neuropsychologist
   - Neurologist
   - Geriatrician
   - Case manager
   - Other, namely:
4. **How many years of work experience do you have in the outpatient clinic setting?**
5. **How many new patients do you see on average per week?**
6. **How many consultations do you have on average with a patient?**
7. **How long does a consultation with a patient last on average?**
8. **How long does a patient journey on average last at your memory clinic?**
9. **Did you see the HiG poster?**
   - Yes
   - No
   - I do not remember
10. **Did you see the HiG video for patients?**
    - Yes
    - No
    - I do not remember
11. **Did you see the HiG video for clinicians?**
    - Yes
    - No
    - I do not remember
12. **Did you see the HiG placemat?**
    - Yes
    - No
    - I do not remember
13. **Did you use HiG during your consultations?**
    - Yes
    - No
    - I do not remember
14. **What were your considerations for using/not using HiG during the consultation? Could you elaborate on this?**
15. **To what extent do you think HiG has helped you to get to know patients better?**
    - Not at all
    - A little
    - A lot
    - Very much
16. **Can you please elaborate?** [regarding question 15]
17. **To what extent do you think HiG was easy to use?**
    - Not at all
    - A little
    - A lot
    - Very much
18. **Can you please elaborate?** [regarding question 17]
19. **To what extent do you think HiG has affected your workflow?**
    - Not at all
    - A little
    - A lot
    - Very much
20. **Can you please elaborate?** [regarding question 19]
21. **To what extent do you think HiG has affected the involvement of patients (and care partners) during the consultation?**
    - Not at all
    - A little
    - A lot
    - Very much
22. **Can you please elaborate?** [regarding question 21]
23. **To what extent do you plan to use HiG in consultations in the future?**
    - Very likely
    - Somewhat likely
    - Neither
    - Somewhat unlikely
    - Very unlikely
24. **Can you please elaborate?** [regarding question 23]
17. **How likely is it that you will recommend HiG to a colleague?** 0= not at all likely and 10= very likely

| 0 | 1 | 2 | 3 | 4 | 5 | 6 | 7 | 8 | 9 | 10 |
| --- | --- | --- | --- | --- | --- | --- | --- | --- | --- | --- |

25. **Can you please elaborate?** [regarding question 23]
18. **How likely is it that you will recommend HiG to a patient?** 0= not at all likely and 10= very likely

| 0 | 1 | 2 | 3 | 4 | 5 | 6 | 7 | 8 | 9 | 10 |
| --- | --- | --- | --- | --- | --- | --- | --- | --- | --- | --- |

26. **Can you please elaborate?** [regarding question 23]
27. **Do you have any ideas about factors that hinder or facilitate the implementation of HiG in practice?**
28. **Where do you think would be a good place to embed HiG?**
29. **The following patients participated in the current HiG study [*insert*]. What was their final diagnosis?**
    - Dementia
    - Mild cognitive impairment (MCI)
    - Subjective cognitive decline (SCD)
    - No diagnosis
    - Diagnosis not yet known
    - Other, namely: ……

**Appendix 8. Coding trees**

Consults

| **Code System** | **Frequency** |
| --- | --- |
| Code System | 134 |
| feedback poli day UX | 1 |
| feedback recruitment | 1 |
| recommendation for future development | 2 |
| usability | 17 |
| satisfaction | 14 |
| effective | 1 |
| efficiency | 2 |
| don't know what to fill in on back side | 1 |
| lay-out/design | 1 |
| suitability cards | 3 |
| moment in time | 3 |
| making a choice | 11 |
| difficult | 3 |
| attitude towards HiG | 43 |
| good conversation due to good relationship with clinician | 1 |
| did not select cards | 2 |
| HiG does not help clinician to get to know pt as a person | 3 |
| HiG helps to express your feelings and thoughts during a conversation | 1 |
| HiG provides structure for a conversation | 4 |
| HiG makes you think | 3 |
| HiG facilitates interaction | 2 |
| good relationship with clinician which is reflected in the use of HiG | 1 |
| HiG helps to discuss topics that would not have been discussed otherwise | 3 |
| HiG makes conversatione easier | 2 |
| added value | 5 |
| potential repeated use | 2 |
| maybe with GP | 1 |
| repeated use depends on health situation | 1 |

Semi-structured interviews with people with cognitive complaints

| **Code System** | **Frequency** |
| --- | --- |
| Code System | 134 |
| feedback poli day UX | 1 |
| feedback recruitment | 1 |
| recommendation for future development | 2 |
| usability | 17 |
| satisfaction | 14 |
| effective | 1 |
| efficiency | 2 |
| don't know what to fill in on back side | 1 |
| lay-out/design | 1 |
| suitability cards | 3 |
| moment in time | 3 |
| making a choice | 11 |
| difficult | 3 |
| attitude towards HiG | 43 |
| good conversation due to good relationship with clinician | 1 |
| did not select cards | 2 |
| HiG does not help clinician to get to know pt as a person | 3 |
| HiG helps to express your feelings and thoughts during a conversation | 1 |
| HiG provides structure for a conversation | 4 |
| HiG makes you think | 3 |
| HiG facilitates interaction | 2 |
| good relationship with clinician which is reflected in the use of HiG | 1 |
| HiG helps to discuss topics that would not have been discussed otherwise | 3 |
| HiG makes conversatione easier | 2 |
| added value | 5 |
| potential repeated use | 2 |
| maybe with GP | 1 |
| repeated use depends on health situation | 1 |

Semi-structured interviews with clinicians

| **Code System** | **Frequency** |
| --- | --- |
| Code System | 346 |
| implementation | 46 |
| how to link to electronic patient files | 1 |
| environment/recycling | 3 |
| do (not) develop digital version | 3 |
| costs | 2 |
| what happens when you do not send HiG in advance | 2 |
| time invested by researcher | 2 |
| support when using HiG | 5 |
| doubt if difficult consultation clinician thinks about using HiG | 1 |
| try to balance influence care partner | 1 |
| avoid that it is 'too much' | 3 |
| continuous implementation in hospital and casemanager setting | 1 |
| who should own the tool | 8 |
| professional organisations | 2 |
| NGN | 2 |
| education | 1 |
| poli | 3 |
| casemanagers | 2 |
| supervisor with co-assistants | 1 |
| recommended use based on story of patient | 5 |
| recommended when diagnosis is clear | 2 |
| recommended after establishing a relationship with someone | 3 |
| recommended when implementation and borging is clear | 1 |
| not recommended in research setting | 1 |
| not recommended due to limited experience with HiG (n=1) | 6 |
| not recommended when someone is an open book | 2 |
| only when difficult situation | 1 |
| already lot of questionnaires and lists | 2 |
| logistics are too much | 11 |
| when and who should use HiG | 12 |
| impact on workflow | 15 |
| using HiG in consult takes extra time | 5 |
| to respond properly to cards takes time | 2 |
| i did frame the consultation time | 2 |
| HiG may surprise you as you don't make the agenda | 1 |
| overlap with regular topics | 8 |
| care partners often address medical topics | 1 |
| if extra time due to discussion certain topics only good for purpose of consult | 1 |
| no extra time during consultation | 2 |
| usability | 29 |
| barrier may be that people have to prepare at home | 1 |
| practical and concrete | 1 |
| visibly seeing cards helps | 3 |
| not for everyone | 1 |
| little work for clinician | 1 |
| aesthetics & design | 4 |
| too complex | 4 |
| too many cards | 1 |
| easy to put cards on the table | 2 |
| easy to use | 7 |
| satisfaction | 8 |
| Effective | 7 |
| attitude towards HiG | 40 |
| HiG facilitates conversation between patient and care partner | 1 |
| HiG helps patients to prepare | 3 |
| patients seemed grateful for being able to discuss HiG cards | 2 |
| HiG helps patients to feel seen and heard | 1 |
| patients initiate topics due to HiG | 1 |
| HiG changes the patient-clinician relationship in a positive way | 1 |
| HiG helps to discuss topics that would not have been discussed otherwise | 4 |
| HiG does not help to improve involvement of patients during consultation | 2 |
| helps to add focus to a consultation | 3 |
| HiG does not necessarily help to deepen conversations | 9 |
| HiG helps to get to know patients | 5 |
| more than expected | 1 |
| deepened our relationship | 2 |
| next time we can refer back to this consultation | 1 |
| especially useful with 'difficult' conversations | 6 |
| HiG helps to improve involvement of patients during consultation | 7 |
| acknowledges need for idea of HiG | 5 |
| psychosocial support is part of providing good care | 1 |
| topics are important for patients | 2 |
| added value | 1 |
| proactive care planning | 1 |
| for patient autonomy | 1 |
| for shared decision-making | 2 |
| patients do not tell everything | 1 |
| time pressure | 1 |
| pressure to 'present well' to a doctor | 1 |

**Appendix 9. Consolidated Criteria for Reporting Qualitative Research (COREQ)**

*see separate PDF*
